# Metabolomics reveals novel insight on dormancy of aquatic invertebrate encysted embryos

**DOI:** 10.1038/s41598-019-45061-x

**Published:** 2019-06-20

**Authors:** Evelien Rozema, Sylwia Kierszniowska, Oshri Almog-Gabai, Erica G. Wilson, Young Hae Choi, Robert Verpoorte, Reini Hamo, Vered Chalifa-Caspi, Yehuda G. Assaraf, Esther Lubzens

**Affiliations:** 1 Natural Products Laboratory, Institute of Biology, Leiden University, Leiden, The Netherlands; 2 metaSysX GmbH, 14476 Potsdam-Golm, Germany; 3 Faculty of Biology, Technion, Haifa, Israel; 4 College of Pharmacy, Kyung Hee University, 02447 Seoul, Republic of Korea; 5 National Institute for Biotechnology in the Negev, Ben-Gurion University of the Negev, Beer-Sheva, Israel; 6 The Fred Wyszkowski Cancer Research Lab, Technion, Haifa, Israel

**Keywords:** Metabolomics, Ecophysiology

## Abstract

Numerous aquatic invertebrates survive harsh environments by displaying dormancy as encysted embryos. This study aimed at determining whether metabolomics could provide molecular insight to explain the “dormancy syndrome” by highlighting functional pathways and metabolites, hence offering a novel comprehensive molecular view of dormancy. We compared the metabolome of morphologically distinct dormant encysted embryos (resting eggs) and non-dormant embryos (amictic eggs) of a rotifer (*Brachionus plicatilis*). Metabolome profiling revealed ~5,000 features, 1,079 of which were annotated. Most of the features were represented at significantly higher levels in non-dormant than dormant embryos. A large number of features was assigned to putative functional pathways indicating novel differences between dormant and non-dormant states. These include features associated with glycolysis, the TCA and urea cycles, amino acid, purine and pyrimidine metabolism. Interestingly, ATP, nucleobases, cyclic nucleotides, thymidine and uracil, were not detected in dormant resting eggs, suggesting an impairment of response to environmental and internal cues, cessation of DNA synthesis, transcription and plausibly translation in the dormant embryos. The levels of trehalose or its analogues, with a role in survival under desiccation conditions, were higher in resting eggs. In conclusion, the current study highlights metabolomics as a major analytical tool to functionally compare dormancy across species.

## Introduction

Dormancy is a complex physiological state that facilitates the survival of organisms during unfavorable environmental conditions and is characterized by an arrest of development and in most cases it is associated with significant metabolic alterations and adaptations. Development and metabolism are resumed by species-specific cues, indicating restoration of favorable environmental conditions. Dormancy occurs in a large number of evolutionary unrelated organisms - from prokaryotes to mammals^1,2^. Despite numerous publications in the past decades, we still lack information on how organisms enter and exit from dormancy and how the latter state is maintained. The term dormancy refers here to a long-term phenomenon and differs from an annual or periodic event, which characterizes insect diapause. In insects, diapause is a genetically-programmed response and involves endocrine, neuroendocrine, metabolic, molecular, cellular, enzymatic and behavioral changes^3,4^. Dormancy or diapause may occur at any life developmental stage: egg, embryo, larvae or adults. Developmental arrest at embryonic developmental stages is well known in aquatic invertebrates such as Cladocera (*Daphnia* species), the brine shrimp *Artemia*, copepods and rotifers^5^, insects^4^, vertebrates such as fish^6^ as well as mammals^7–11^. Interestingly, artificially induced diapause in mouse embryos (or paused blastocysts) serves in embryonic cell pluripotency studies^12^. In many aquatic invertebrates exhibiting cyclic parthenogenesis, induction of sexual reproduction leads to the formation of dormant encysted or encased embryos in the form of resting eggs (RE, e.g. rotifers, copepods), cysts (e.g. *Artemia* sp.) or ephippia (e.g. *Daphnia* sp.).

In organisms where dormancy is associated with desiccation, there is a formation of a glassy or vitrified state, which entails an increase in cellular viscosity and leads to a dramatic reduction in biochemical reactions, including metabolism and other functional pathways^13^. A few developmental stages and/or adult invertebrate species survive desiccation for numerous years, including nematodes, bdelloid rotifers and tardigrades^14–16^, rotifer RE^17–20^ and insects^21–24^. Prior to desiccation in various organisms, numerous metabolites accumulate that may ameliorate the harmful effects of dehydration, including sugars such as glucose and trehalose, polyols as mannitol, erythritol and glycerol, as well as amino acids^5,25–28^. The role of trehalose in desiccation has been disputed. In numerous desiccated organisms, trehalose has been associated with the formation of the glassy state (especially in *Artemia* cysts, where it constitutes 15% of the dry weight)^29^. However, it was not detected in desiccated bdelloid rotifers and tardigrades^16,30^. Only small amounts of trehalose (0.35% of the dry weight) were found in rotifer RE^31^, although two transcripts encoding for trehalase-6-phophate synthase, and four trehalose phosphate synthase enzymes, were detected in rotifer RE^17,32^, suggesting its biosynthesis in rotifers.

There are, however, several examples of survival for decades or even centuries in a non-desiccated state in aquatic organisms such as rotifers, cladocerans or copepods, in the form of RE or ephippia^33–38^. Most amazing is the survival of *Daphnia* ephippia in a lake sediment cores for over six centuries^39^. The encased or encysted dormant embryos or eggs differ morphologically from the non-dormant embryos or eggs. This observation suggests an accumulation and enrichment of specific compounds that can support long-term dormancy^40^.

Surprisingly, similar phenotypes and common functional pathways have been identified, regardless of the diversity and complexity in the survival strategies of organisms displaying dormancy. These include repression of metabolic pathways, attenuation of cell cycle progression, changes in carbohydrate and lipid metabolism, resistance to stress and protection of cellular structures^1,4,29,41,42^. Numerous studies describe transcripts and proteins in association with dormancy but there is little transcriptional similarity among dormancies across species^43^ and diverse transcriptional strategies for producing them were reported in insects^44^ (and references cited in this publication). The similarity in phenotypes can be partially explained by proteome profiling, which shows a low correlation with transcriptomics^17^. Metabolomics holds promise to offer additional information on dormancy as metabolites are downstream of gene transcripts and have a key impact on protein expression^45,46^.

A search using metabolomics on changes associated with the “diapause syndrome” has been reported for numerous insect species but the number of the identified metabolites is relatively low, ranging from 20 to 75 and therefore were not associated with full functional pathways^47–56^. Very few studies examined dormant and non-dormant eggs of aquatic species; these include a chemical composition study on subitaneous and diapause eggs of a copepod^57^ and of *Daphnia*^58^, but not on *Artemia* or rotifers.

Metabolomics is an emerging field concerned with the study of the organism’s physiological state at the metabolite level by offering means to obtain a comprehensive view of the changes in abundance of numerous low molecular weight compounds simultaneously (see the Introduction sections in^48^ and^59^). In parallel to the transcriptomics and proteomics, the set of metabolites synthesized by an organism constitute its metabolome^45^. The metabolites are the products of cellular processes and in comparative experiments metabolomics analyses define importance by the relative changes in metabolite abundance. Changes in metabolites can reveal biomarkers of the integrated response of an organism. Most importantly, metabolomics is applicable to all species without any prior knowledge of the genomic sequence^52^ and sampling is accomplished by extracting metabolites. The most common methods of analysis of the metabolites is by chromatographic analysis [gas chromatography/mass spectroscopy (GC-MS), liquid chromatography/mass spectrometry (LC-MS) and MS/MS)] or by nuclear magnetic resonance (NMR) spectroscopy. The large datasets obtained are subjected to principal components analysis (PCA) or other chemometric tools. Using this metabolomics approach, the present metabolites can be related qualitatively and quantitatively to the different physiological states and various treatments. The disadvantages of LC-MS and GC-MS, as well as MS or MS/MS, is the difference in detector response for metabolites, which means that for every single compound, relative changes between the different experimental conditions can be measured, but quantification is only possible by using a calibration curve for each single compound. All metabolomics methods have a major limitation in the identification of the compounds. For well-known compounds, direct comparison with standard compounds is possible, but for novel or rare compounds this represents a major problem. The advantages lie in the rather unbiased nature of the data gathering following strict protocols enabling the comparison of the results over many years and in between laboratories. The handling of big data with different chemometric/statistic tools allows the comparison of “omics” data, generating novel hypotheses in this systems biology type of approach. Even though one should keep in mind that living systems have four dimensions (three of space and one of time), a metabolic flux through biochemical pathways cannot be confidently deduced from metabolomics data, which only describes the whole organism at a single time point.

The aim of the current study was to determine whether metabolomics provides additional functional insights into explaining the “dormancy syndrome” by underscoring functional pathways and metabolites, enhancing a more comprehensive view of this phenomenon. Towards this end, we herein used the rotifer dormant embryos (also known as RE) as a model. We compared metabolomics profiles of dormant and non-dormant eggs (also known as amictic eggs, AM) of the rotifer *Brachionus plicatilis*. Proton nuclear magnetic resonance (^1^H NMR) spectroscopy and high-performance liquid chromatography coupled to a diode array detector (HPLC-DAD) were used in initial experiments. Afterwards, lipophilic and polar metabolites of dormant and non-dormant rotifer eggs and algae (*Nannochloropsis* sp.) fed to the cultured rotifers were analyzed with the use of LC-MS and GC-MS. The relatively large number of identified features facilitated a functional allocation to numerous biochemical pathways. The changes in features in these pathways suggest explanations for the reduced metabolism, lack of transcription and translation during dormancy. Moreover, features with putative functions in signal transduction pathways were also identified. Since rotifer RE are tolerant to desiccation^17–20,37^, we also searched for small molecules, with a possible role in the formation of a glass and protection of membranes and proteins. The current study demonstrates the functional importance of metabolomics in complimenting transcriptomics and proteomics in deciphering the molecular basis underlying dormancy. Our findings provide a novel view for explaining the long-term survival of dormant embryos.

## Results and Discussion

**Figure 1:**
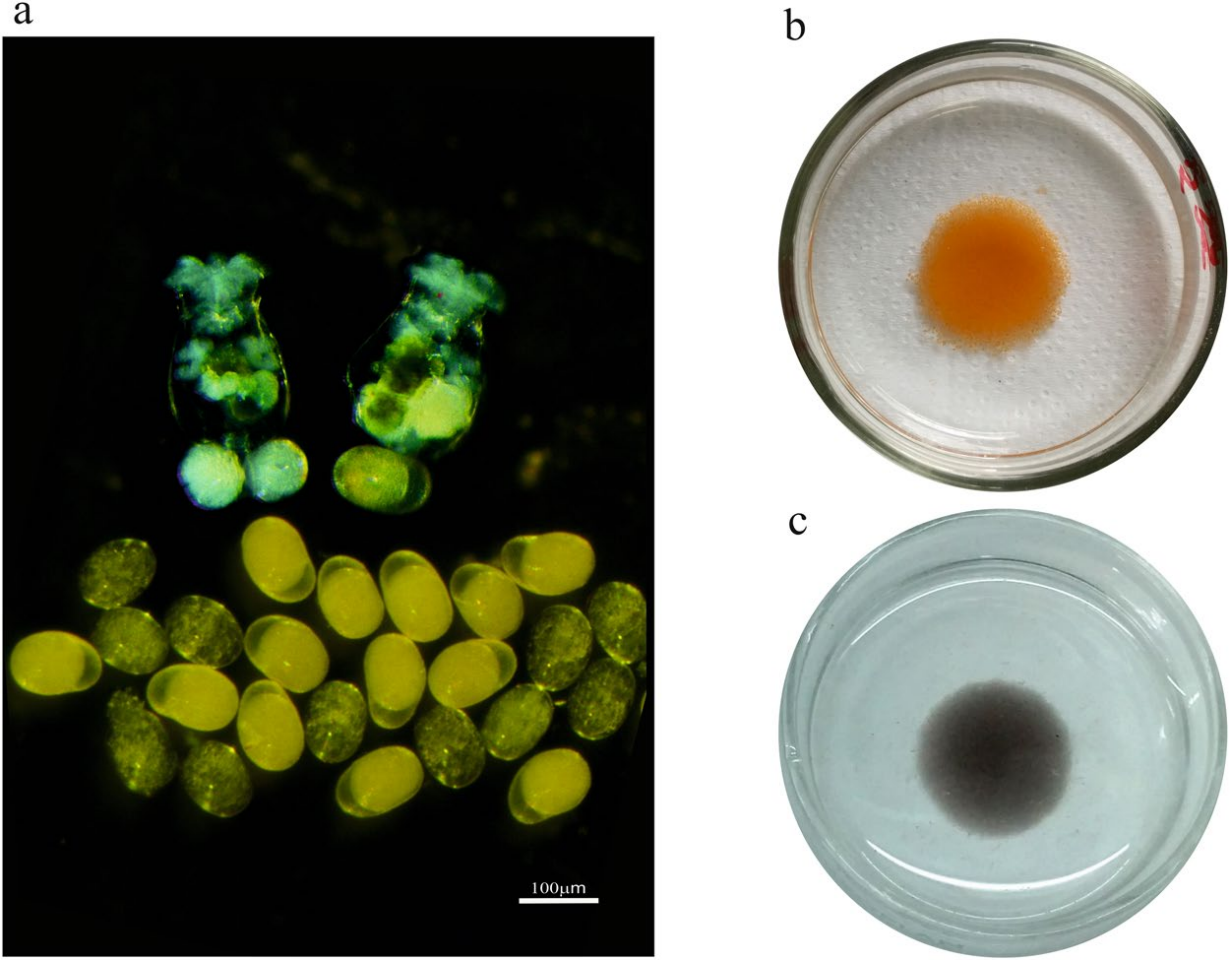
**(a)** Female rotifers carrying non-dormant amictic eggs (AM) or embryos (top left) and a dormant encysted embryo or resting eggs (RE; top right). Transparent AM and yellow colour RE are shown below the females. **(b)** Cleaned RE (~50,000) in a 35 mm glass petri dish. **(c)** Cleaned AM (~40,000) in a 35 mm glass petri dish.

Morphological differences observed between AM and RE that were carried by females are depicted in Fig. 1, suggesting possible differences in their contents of metabolites. RE display a brown/orange colour that was absent in AM. Therefore, these two types of eggs were subjected to metabolomics studies as outlined in Fig. S1. Both egg types were hydrated with a similar percent of moisture content (83.9 ± 2.2% and 82.8 ± 0.7%, Mean ± S.D. N = 3; for AM eggs and RE, respectively).

### ^1^H NMR spectral analyses

**Figure 2:**
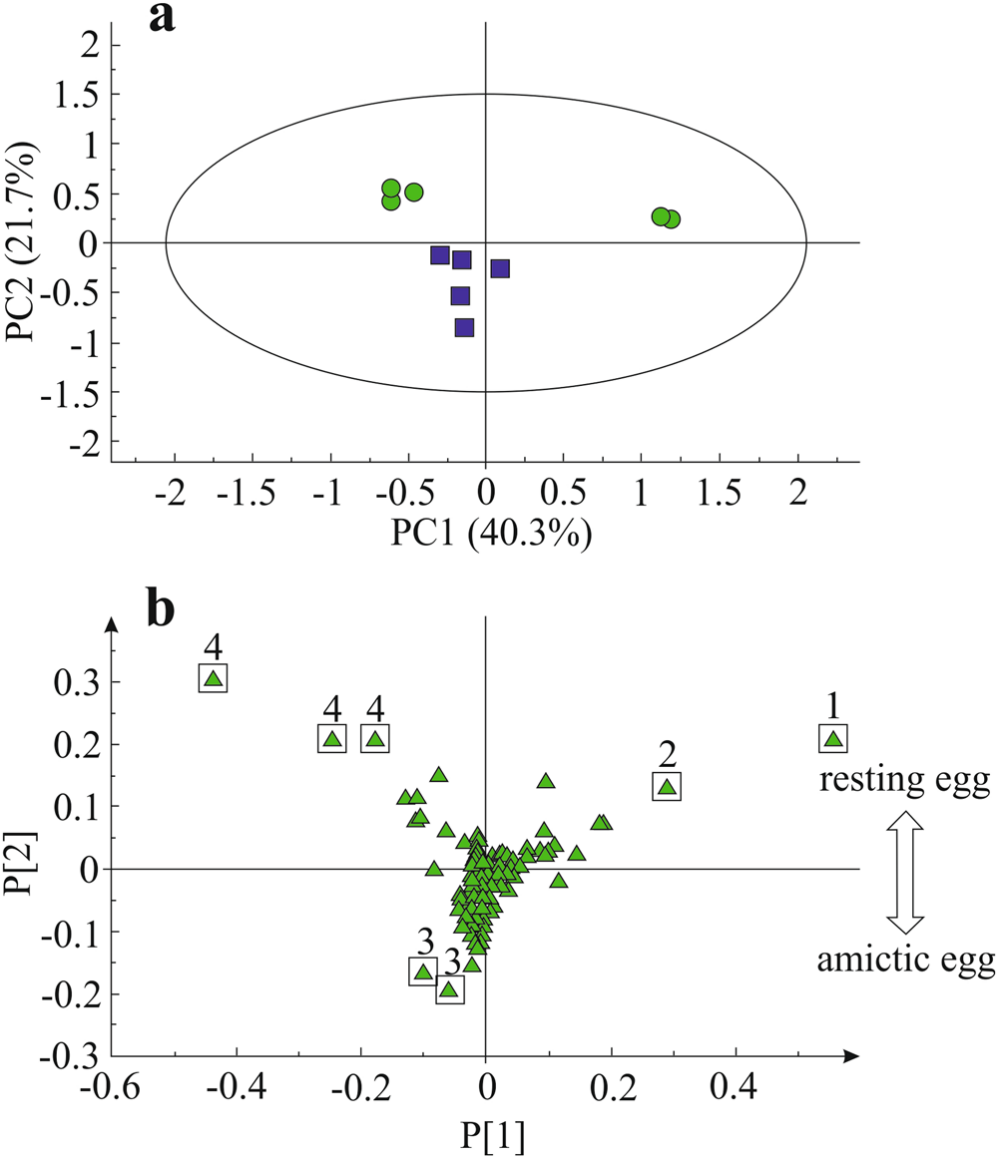
The principal component analysis (PCA) score plot **(a)** and loading plot **(b)** using the first two principal components derived from ^1^H NMR spectra data sets of AM (blue squares) and RE (green circles; *n* = 5). 1. Trehalose; 2. CH_2_ signals of lipids and fatty acids, 3. Choline, 4. Creatine-like compound.

The ^1^H NMR spectra of AM and RE were calibrated to the chemical shifts of trimethylsilylpropionic acid (TSP). A preliminary analysis of the ^1^H NMR spectra showed a lower abundance of metabolites in RE (Fig. S2a,b) in comparison with AM eggs. For identification from ^1^NMR signals, in-house reference database and literature references were used. The identification was based on matches of the chemical shift of the peaks and their splitting patterns with references displaying a confidence level of 99.5–99.9%. Mass spectrometric identification was based on a matching mass, retention time and mass spectrum with references exhibiting a confidence level of at least 95%. Among the detected metabolites were amino acids (valine, alanine, glycine, glutamic acid and homoserine), acetate, acetone, betaine, trehalose, proline, adenosine monophosphate (AMP) and creatine-like compounds (Fig. S2a–g)). Binned NMR spectral data of AM (n = 5) and RE (n = 5) were subjected to principal component analysis (PCA) as shown in Fig. 2a. In this PCA, the first three principal components (PCs) accounted for 77.8% of the total variance of the data (binned NMR data used for discriminating the NMR profiles by PCA are not shown). The ^1^H NMR spectral data representing one sample each of AM and RE were separated by principal component 2 (PC2, namely 21.7%). The PCA loadings plot (Fig. 2b) and the contribution plot (not shown) based on PC1 and PC2, allowed the identification of potentially important metabolites in these two egg types. Signals were highlighted for the following compounds: choline (3.20/3.24 ppm), lipids and fatty acid (CH_2_, 1.28 ppm) and a creatine-like compound (3.28, 3.84 ppm). Extraction of the metabolites starts with the same ratio of dry material and solvent. This means that when most metabolites in RE are present at lower amounts than in AM (Fig. S2), the percentage of the extracted metabolome of the total biomass is lower in RE. Consequently, a larger fraction of the RE biomass is not analyzed by the NMR, this could be due to the presence of poorly soluble compounds (e.g. lipids, polysaccharides, and certain proteins). To identify pigments in RE, targeted analysis of lipids was performed by HLPC-DAD of the xanthophylls and by GC-MS. Two major xanthophylls were detected at 440 nm only in the AM (Fig. S2h,i) at low quantities.

### Global metabolome profiling by LC-MS, MS/MS and GC-MS measurements

#### General information

**Figure 3:**
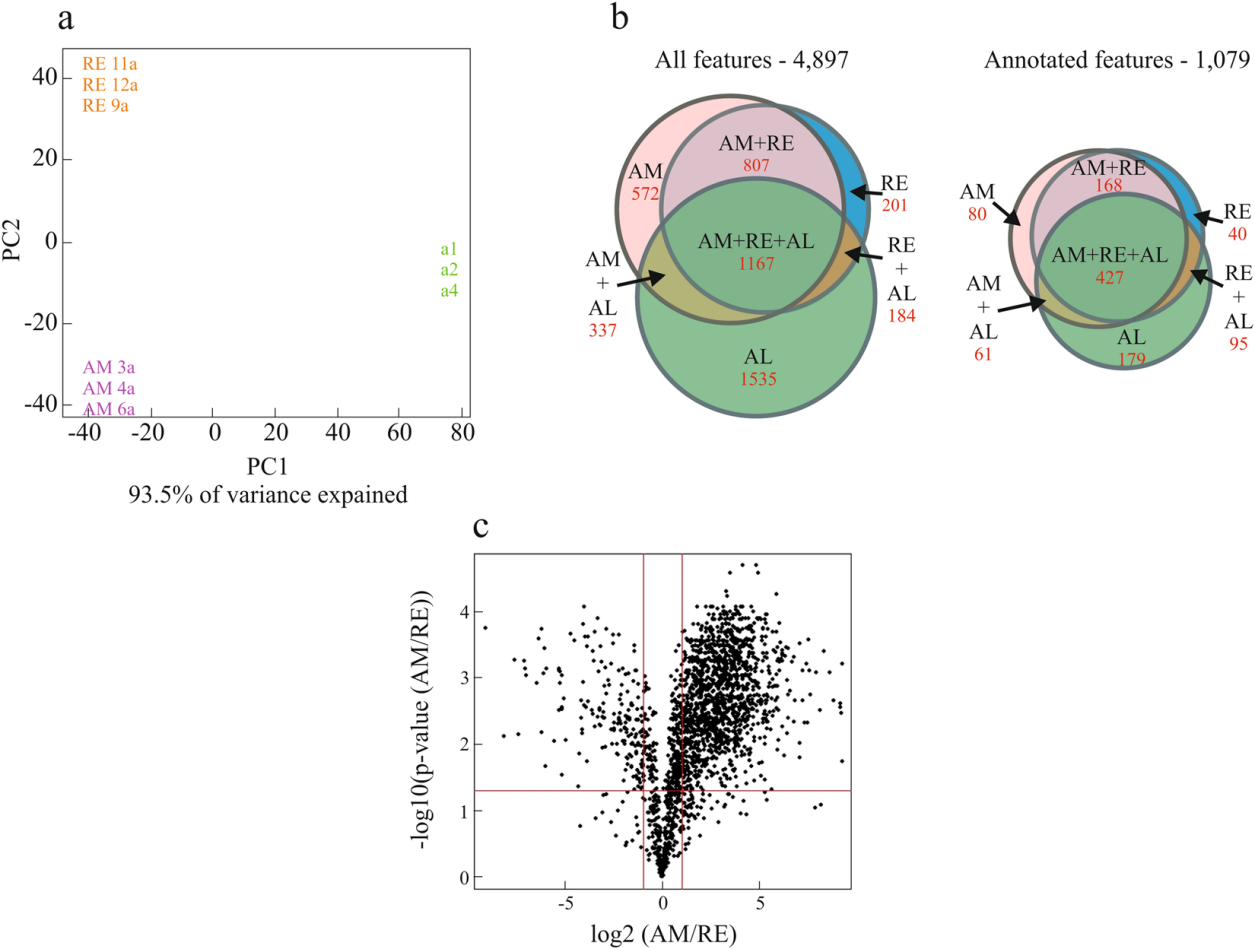
A comparison of the features obtained by LC-MS, MS/MS and GC/MS analyses for AM, RE and algae. (**a**) PCA score plot on normalized data showing differences in the features of AM, RE and algae (*Nannochloropsis* sp.). Samples from three cultures were used for each egg type (AM or RE) and three technical samples for algae (**a**). (**b**) A Venn diagram showing the shared and non-shared features of algae (AL), dormant (RE) and amictic eggs (AM). The diagrams for all the features and for the annotated features are shown on the left and on the right, respectively. The features for AM or RE are based on their occurrence in all three replicate samples of each egg type. (**c**) A Volcano plot showing logarithmically-transformed fold-change (base 2) and negative logarithm of *p*-value (base 10) (−log10 of p value) of AM vs. RE per feature in a student t test using normalized intensity values. The red cutoff lines indicate a logarithmically transformed Fold-Change of 2 (log2 = 1) and an FDR adjusted *p*-value of 0.05.

A total of 4,897 features were observed (Table S1; normalized data) of which 1,079 were annotated to known features (22%). Approximately 63.3% and 54.9% of the annotated features were found in AM and RE, respectively, and 70.0% were found in the algae that served as food for the rotifers. The total ion chromatograms showed large differences between the RE and AM samples. Two methods were used for analyses of data (see Materials and Methods for details): (a) Median group-wise normalization (Fig. S3a,b; Table S1) and (b) Data was not normalized (Fig. S4a,b). Globally, AM eggs showed higher abundance of features in comparison with RE (Fig. S4a; un-normalized data, Table S2) but differences were smaller for the annotated features (Fig. S4b; un-normalized data, Table S2). A high correlation was found for the fold-change values in the comparison between the normalized and unnormalized data (Tables S1, S2 and Fig. S5). Unless otherwise indicated, the Figures and Tables in the current paper use the normalized dataset. PCA demonstrates the difference between the two egg types and the algae, with 93.5% of the variance explained by PC1 on PC2 (Fig. 3a) and 68.6% by PC1 on PC3 (data not shown). A relatively large number of features was shared by AM, RE and algae (Fig. 3b). Of the annotated features, 80 were detected only in AM, 40 were detected solely in RE and 179 in algae. The features that were detected only in RE, included triacylglycerols, phospholipids, dipeptides, L-cysteic acid, creatine and guanidineacetic acid, a precursor for creatine (http://www.hmdb.ca/metabolites/HMDB0000128; Table S4). The features that were detected only in AM included ceramides (see the discussion below), diacylglycerols, triacyglycerols, uridine diphosphate with a function in glycogenesis^60^, the neurotransmitter dopamine and most notably adenosine triphosphate (ATP) (Table S4). Of the 168 annotated features that were shared by AM and RE (and not present in algae), 143 were lipids. Interestingly, a higher abundance (depicted as fold change) of features in AM in comparison with RE was highlighted by a Volcano plot (Fig. 3c; Table S1, Normalized data all features), as found in the NMR analyses. Of the annotated features, 503 features differed significantly (FDR adjusted p value < 0.05) between AM and RE, with 134 features showing a fold-change >10, and 405 features showing a fold-change >2 between AM vs RE. The list of 10 features with the highest fold-change values in the comparison of AM versus RE or RE versus AM is shown in Tables 1 and 2. Most notable were L-cystine with a fold-change >1,250 and ascorbic acid with a fold-change >950 (Table 1). A few dipeptides showed a higher abundance in RE vs AM (Table 2, Table S1). Cluster analysis revealed (Fig. S6) a large number of features showing higher abundance in AM, as found in the NMR analysis and as reported above. Numerous features were only found in AM or RE but not in algae (white lines in Fig. S6), most of which were lacking annotations. A few features show higher abundance in RE, consisting mainly of dipeptides, phosphatidylcholine (PC) and phosphatidylethanolamine (PE, in the upper part of the heat map) as well as sugars including trehalose, turanose, maltotriose, and triacylglycerols (TAGs, in the bottom part of the heat map; Table S3).

**Table 1:** A list of the 10 features with the highest fold change (higher in AM) (in the comparison of amictic (AM) and resting eggs (RE) (t- test at FDR-adjusted p < 0.05, Table S1).

Peak ID	Name of Compound	Fold change (AM vs RE)
GC_128	Cystine	1250.29
PN_1374	Ascorbic acid	994.95
PP_22458	Adenylosuccinic acid	322.20
PP_5097	Uric acid	280.15
GC_142	Maltose	135.52
GC_63	Nicotinamide	107.95
GC_125	Glucose 6-phosphate	102.84
PP_3563	Acetylcholine	90.32
PN_632	Malic acid	81.30
PP_552	(R)-2-Aminobutanoic acid|3-Aminoisobutyric acid	71.58

**Table 2:** A list of the 10 features with the highest fold change (higher in RE) in the comparison of resting eggs (RE) and amictic (AM) eggs (t- test at FDR-adjusted p < 0.05, Table S1).

Peak ID	Name of Compound	Fold change (RE vs AM)
GC_158	Unknown, similar to Trehalose	61.66
PP_10295	Aspartyl-L-leucine	38.44
GC_144	D-Trehalose	36.35
LP_4371	LysoPC (FA 18:1)	31.68
PP_9280	L-leucyl-L-valine	26.61
PP_11069	L-leucyl-L-glutamic acid	22.63
PP_8501	L-seryl-L-leucine	19.59
LP_7477	PC 36:2	16.93
PP_2768	L-Aspartic acid/Iminodiacetic acid	9.02
PP_3561	4-Guanidinobutyric acid	8.79

#### Metabolites with specific interest

**a)** *Carbohydrates:* Surprisingly, numerous carbohydrates (sugars and polyols) that were associated with diapause or resistance to desiccation in other organisms were significantly more abundant in AM in comparison with RE (Table 3). Six features were identified with the names “D-Trehalose” (two features), “Unknown similar to Trehalose” (three features), “D-Gentiobiose|D-Maltose|D-Trehalose|Turanose” (one feature), with fold change values ranging from 1.37 to 61.66, in the comparison of RE versus AM (Table S1). As discussed in the Introduction, resistance to desiccation may be related to the presence of various sugars and polyols (e.g. trehalose, *myo*-inositol), organic acids and bases (e.g. betaine, creatine), including amino acids. Choi *et al*.^61^ hypothesized that these compounds may form Natural Deep Eutectic Solvents (NADES) that can strongly retain water and form a medium in which proteins can be conserved. Some candidates for these NADES were at higher abundance in AM in comparison with RE. Trehalose, with an established role in the formation of a glass at ambient temperatures was previously reported to occur at low levels in rotifer RE^31^; transcriptome and proteome profiling studies revealed the occurrence of enzymes in the trehalose synthesis pathway^17,32,62^. Our current results generally indicate a higher abundance of trehalose in RE, suggesting a functional role for this metabolite in dormant RE, in contrast to a previous publication^31^. Alternatively, trehalose may serve as a source of carbohydrates for metabolism in rotifers as in insects (reviewed previously^63^). Another possible candidate for forming the glassy state during desiccation in rotifer RE could be attributed, as in tartigrades^16^, to late embryogenesis abundant proteins. These intrinsically disordered proteins were identified in high abundance in RE^17,64,65^. Rotifer RE survive desiccation after a short (15–20 min) dehydration period^17^, therefore, it can be assumed that all the components that facilitate long-term survival in the dry form are within the RE at the end of their formation (between 2–5 days). It remains to be determined whether or not these components also contribute to the survival in the wet state as RE can be kept for at least 21 years in the lab in this state^32^.

**Table 3:** A list of carbohydrates in rotifer eggs with an association to diapause or tolerance to desiccation in organisms (Table S1).

Name of feature	FDR p value (AM vs RE)	FC (AM vs RE)
Maltose	0.016	135.52
Galactose	0.011	6.21
glucose	0.001	5.24
Ribose	0.006	5.04
Mannitol	0.004	4.06
Fructose/psicose	0.005	3.65
Glyceric acid	0.003	3.24
Saccharic acid	0.052	2.73
Glycerol	0.010	1.27
Trehalose*	0.0001–0.05	0.016–0.23
*myo*-inositol	0.002	9.87

All the features (excluding trehalose) were significantly more abundant in AM in comparison with RE.

*Six features were identified with the names “D-Trehalose” (GC_144), “Unknown similar to Trehalose (GC_137; GC_143; GC_144; GC_158) or “Gentiobiose|D-Maltose|D-Trehalose|Turanose (LN_2338), showing a fold change of RE vs AM, ranging from 1.37 to 61.66.

**b)** *Vitamins:* Vitamins were more abundant in AM than in RE, except for riboflavin which was solely detected in RE. α-tocopherol acetate (vitamin E) showed similar abundance in the two egg types (Table 4), supporting early studies on the role of tocopherol in reproduction of rotifers^66^. Interestingly, astaxanthin, a yellow pigment and an antioxidant, was significantly more abundant in AM in comparison with RE (*p* = 0.009; fold change = 62.37, Table S1). Uridine 5′-diphosphoglucuronic acid which serves as an intermediate in the biosynthesis of ascorbic acid, was at very low detection levels in RE (Table S1).

**Table 4:** Difference in the abundance of selected vitamins between AM and RE (Table S1).

Name of	FDR p values (AM vs RE)	FC (AM vs RE)
Ascorbic acid (vitamin C)	0.019	994.950
Biotin	0.001	5.000
Pantothenic acid (vitamin B5)	0.003	7.281
Riboflavin (Vitamin B2)	Only detected in RE	
α-Tocopherol acetate (vitamin E)	0.357	0.699

Similar abundance of Vitamin E was found in the two egg types.

**Figure 4:**
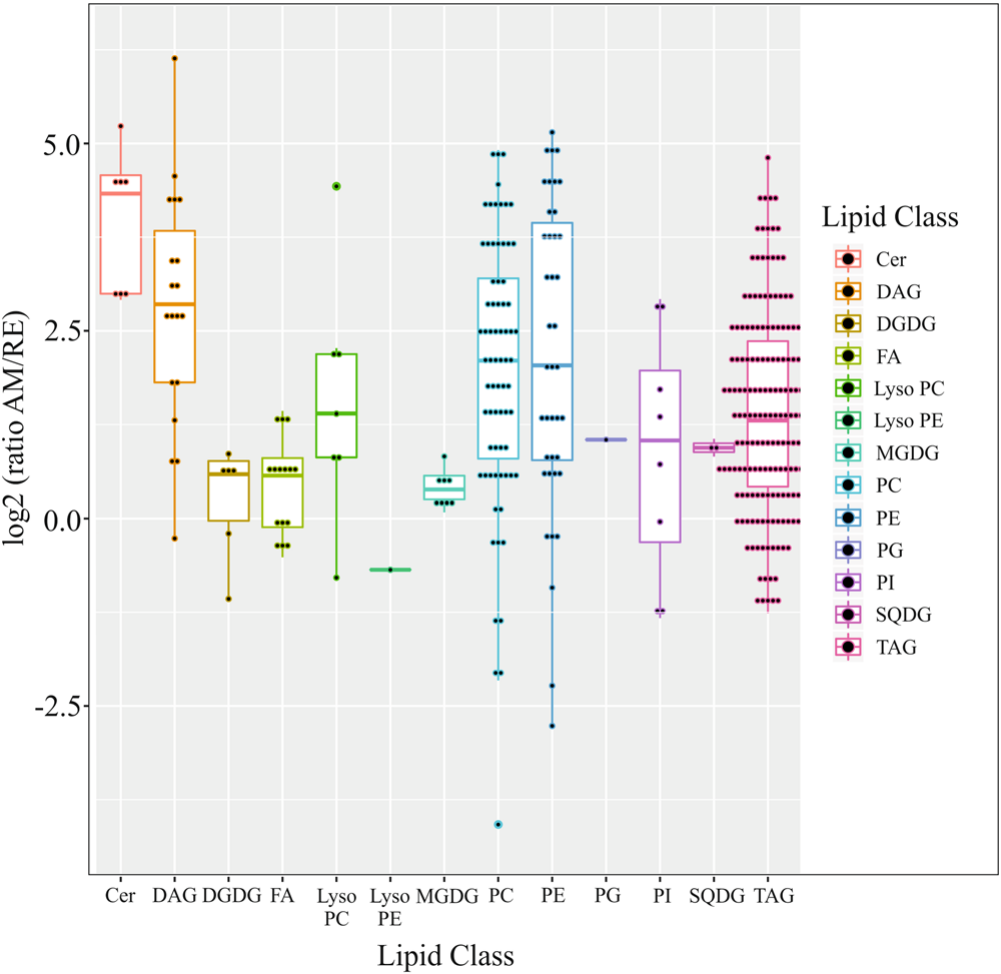
The relative abundance (ratio of AM/RE) of lipid classes in dormant (RE) and non-dormant (AM) embryos. Each fatty acid or lipid is depicted as a dot within each one of the different classes. For example, a large number of different triacylglycerols were identified within TAG. Abbreviations: Cer—ceramide, DAG—diacylglycerol, DGDG—diacylgalactosyl diacylglycerol, Lyso PC—lysophosphatidylcholine, Lyso PE—lysophophatidylethanolamine; MGDG—Monogalactosyl diacylglycerol, PC—Phosphatidylcholine, PE—Phosphatidylethanolamine, PG—Phosphatidylglycerol, PI—Phosphatidylinositol, SQDG—Sulfoquinovosyl diacylglycerol, TAG—triacylglycerol.

**c)** *Lipids:* Relatively many lipids, lipid classes and fatty acids were identified in AM, RE and algae and fatty acid composition was determined or partially determined for 338 features (Table S1). A large variation was found in their relative abundance in AM vs RE as shown in Fig. 4. Most interestingly, ceramides showed higher abundance in AM vs RE (Fig. 4). Ceramides have a central role in sphingolipid biosynthesis and catabolism. Sphingolipids are synthesized *de novo* from serine and palmitate, which condense to form 3-ketodihydrosphingosine through the action of serine palmitoyl transferase (SPT). The generic “ceramide” family is comprised of >50 distinct molecular species, of which about 25 were identified in the current study (Fig. 4 and Table S1). Pharmacological inhibition of SPT extends life span in both *Caenorhabditis elegans*^67^ and yeast^68^ and ceramide glucosyltransferase is involved in oocyte formation and early embryonic cell division in *C. elegans*^69^. Interestingly, SPT was detected in AM but not in RE^17^.

**d)** *Pigment metabolites*. As abovementioned, a clearly visible difference in AM and RE is the yellow-orange colour of the latter (Fig. 1). Astaxanthin (a red coloured pigment) and xanthophyll (a yellow coloured pigment) were detected in RE (Table S1 and Fig. S2, respectively), but their abundance was much lower than in AM. Possibly, the orange-brown colour of RE could be attributed to riboflavin (a yellow/orange coloured B2 vitamin) which was identified solely in RE (Table 4) and/or ferritin, with its red-brown colour. Ferritin is one of the ten most abundant proteins in RE^17^.

**e)** *Metabolites with a function in response to light*. L-Kynurenine, a low molecular weight metabolite that is obtained from tryptophan metabolism was more abundant in AM than in RE (*p* < 0.006; fold change = 12.28, Table S1). This interesting metabolite is an ultraviolet (UV) chromophore found in lens of vertebrates and prevents light below 400 nm from reaching the retina (reviewed in^70^). The lower abundance of kynurenine in RE may indicate a higher sensitivity to UV irradiation in RE. Since RE respond to irradiation by hatching after an obligatory dormant period and most RE hatch if exposed to 250–310 nm light, the lower abundance of kynurenine in RE may facilitate perceiving proper environmental cues including light for hatching^71^. Proteomics analysis^17^ identified four proteins in the tryptophan metabolism pathway (Fig. S7). Tryptophan 2,3-dioxygenase leading to the generation of L-kynurenine, was not detected in RE. However, a higher abundance of kynurenine 3-monooxygenase [EC:1.14.13. 9] leading to the generation of 3-hydroxy-L-kynurenine was found in RE but this metabolite was not detected in the current study. Kynurenine oxoglutarate transaminase, which is involved in the metabolism of L-kynurenine and hydroxyl-L-kynurenine, was less abundant in RE.

#### Putative functional pathways in association with metabolites

**Figure 5:**
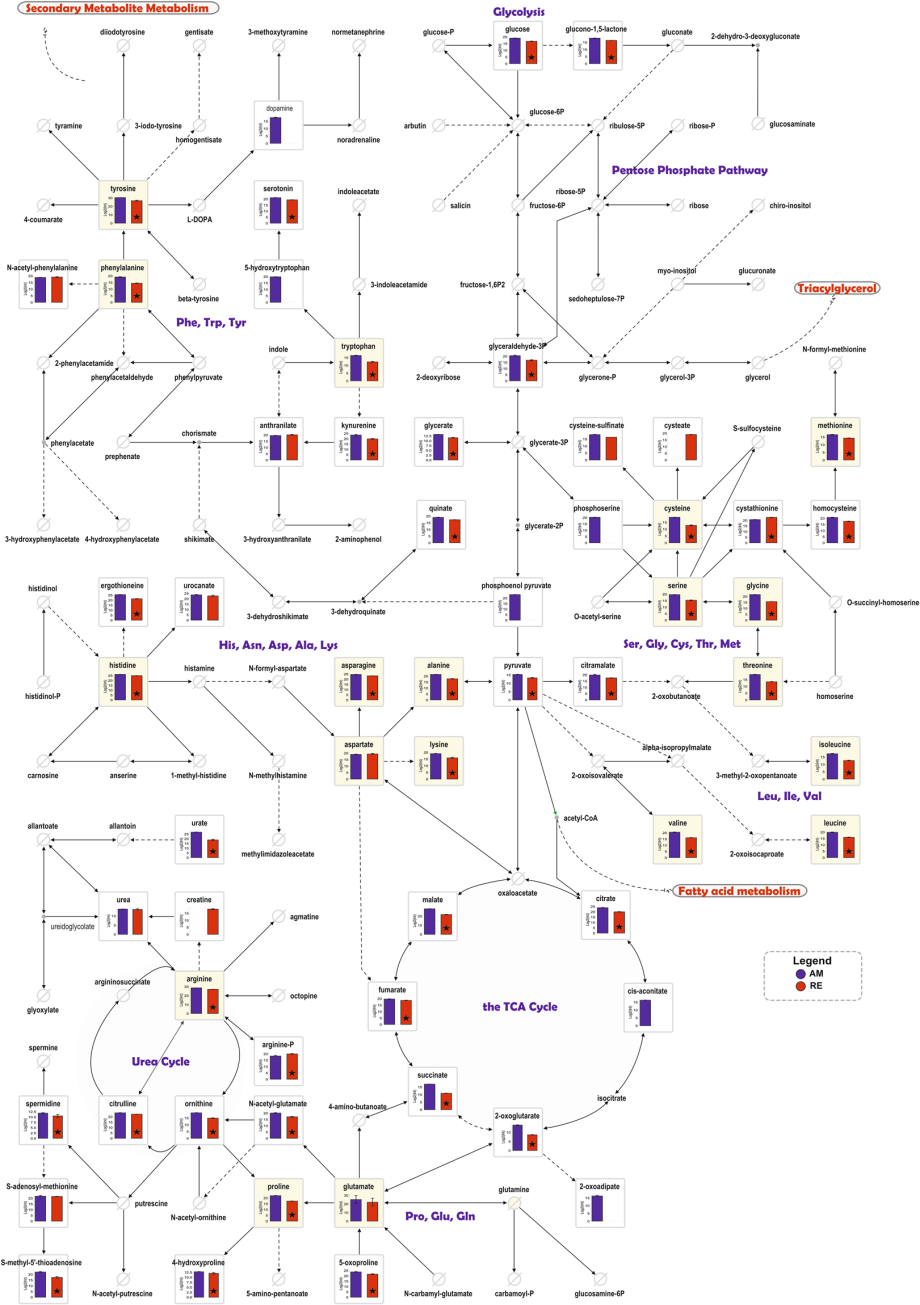
The abundance of features assigned to Glycolysis, Pentose Phosphate pathway, Amino acid metabolism, the TCA cycle and the Urea cycle, in RE (red) and AM (blue). The bars represent the mean values with the S.D. and black asterisks indicate statistically significant differences (*p* < 0.05) between RE and AM. Metabolites that were not detected in these pathways are shown as crossed circles.

**Figure 6:**
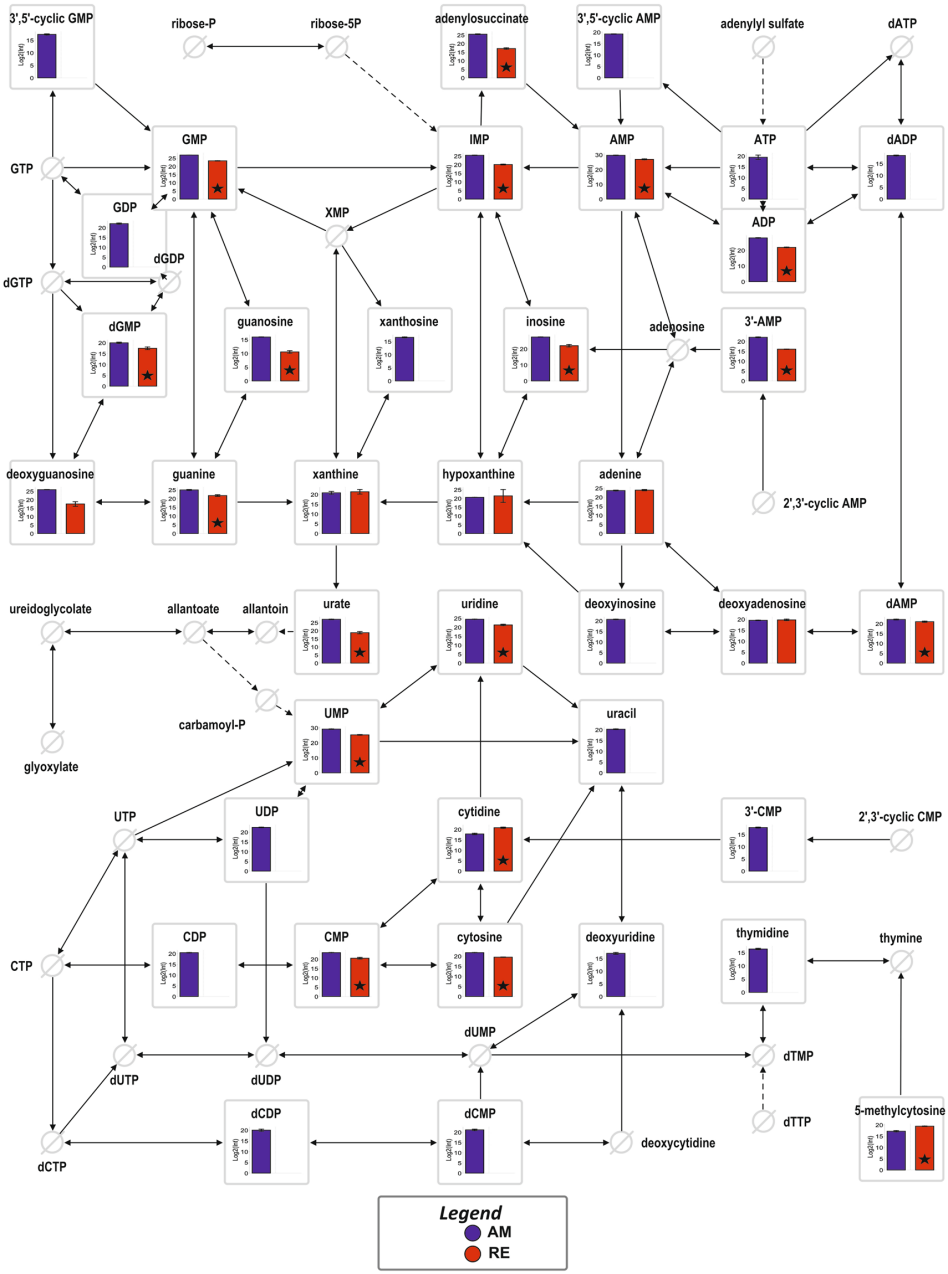
The abundance of features assigned to Purine and Pyrimidine metabolism. The bars show the mean values with the S.D., whereas black asterisks indicate statistically significant differences (*p* < 0.05) between RE and AM. Crossed circles indicate metabolites that were not detected in these pathways.

The relatively large data-set obtained by LC-MS, MS/MS and GC-MS measurements allowed the allocation of features to functional pathways, in a manner similar to high-throughput transcriptomes and proteome studies. These pathways included: Glycolysis, TCA cycle, Urea cycle, Pentose Phosphate Pathway, Amino acid metabolism, Purine metabolism and Pyrimidine metabolism (Figs 5 and 6).

**a)** *Cellular and mitochondrial metabolism:* As depicted in Fig. 5, glucose, glyceraldehyde-3 phosphate and pyruvate were lower in RE. Phosphoenolpyruvate was not detectable in RE. Moreover, TCA cycle metabolites were either lower in RE, or were undetectable at all. These observations suggest a compromised cellular and mitochondrial metabolism in RE. A marked reduction in metabolism is a well-known phenomenon in diapausing insects or dormant organisms^72,73^.

**b)** *The urea cycle****:*** numerous features in this cycle were significantly higher in AM in comparison with RE (Fig. 5), except for creatine, which was not detected in AM, as mentioned before. Creatine is formed from the amino acid arginine and in mammals can be phosphorylated by creatine kinase to form phosphocreatine (PCr), which is used as an energy buffer in skeletal muscles and the brain. In this respect, during times of increased energy demands, ATP can be synthesized rapidly from ADP with the use of PCr through a reversible reaction using the enzyme creatine kinase^74^, which was detected in AM and RE^17^. ADP was found in RE and AM, although it’s relative level in RE is significantly lower than in AM.

**c)** *Amino acid biosynthesis:* The lower abundance of almost all amino acids (excluding aspartate and glutamate) in RE suggests lower translation in RE (Fig. 5; partial information was previously shown^17^).

**d)** *Purine metabolism:* A large number of features in this pathway was either lower or undetectable in RE. These include, most notably, ATP which was not detectable in RE. This is in contrast with *Artemia* cysts, where ATP is not depleted but the concentration of ATP is about five-fold lower compared with post-diapause embryos^75^. *Artemia* cysts placed under anoxia conditions, displayed a rapid decline in the cellular ATP pool which was nearly undetectable^76^, but the state of anoxia in the examined RE has not been determined; they were maintained in air exposed seawater during the period of collection (2–4 days) from the culture flasks. The following metabolites were not detected as well in RE: 3′5′-cyclic GMP, 3′5′-cyclic AMP, GDP, dADP, xanthosine and deoxyinosine (Fig. 6). This is in contrast to *Artemia* cysts, where cAMP and cGMP were identified^77^. These secondary messengers function in intracellular signal transduction pathways, also during diapause or dormancy in many different organisms as previously reviewed^4^. The most plausible mechanism of action is activation of intracellular protein kinases (which in turn phosphorylate and activate enzymes) in response to the binding of membrane-impermeable peptide hormones to the external cell surface^78^. *Artemia* cysts contain massive amounts of guanine-containing nucleotides, with guanosine (5′) tetraphospho (5′) guanosine (Gp_4_G) as the most prominent member of this family. It was suggested that it serves as a source of free energy during anoxia. It may also serve as a source of adenine and guanine nucleotides, due to the apparent absence of the pathway required for the *de novo* biosynthesis of the purine rings in *Artemia* (reviewed in^79^).

**e)** *Pyrimidine metabolism:* in this metabolic pathway too, features were higher in AM than in RE or were undetected in RE (Fig. 6). Most notable were uracil and thymidine, which were not detected in RE and the following metabolites which also were not detected in RE: UDP, 3′ CMP, CDP, dCDP and dCMP. DNA replication and transcription in RE were presumably eliminated as indicated by the absence (or below detection level) of thymidine and uracil, respectively (Fig. 6). Moreover, the nucleobases guanine and cytosine were at significantly lower abundance in RE than in AM but there was no difference in the abundance of adenine between RE and AM. Consistently, in RE, numerous intermediates in the biosynthesis of cytosine and thymidine were either lacking, at low levels or below the detection level. An interesting relationship showing a complicated concordance between the abundance of proteins and features in the purine and pyrimidine metabolic pathways is depicted in Figs S8 and S9.

#### Compounds with a function in promoting or preventing diapause in insects

The naturally occurring diamine, putrescine, plays a role in diapause induction and intensity in insects by enhancing juvenile hormone secretion^80^. Putrescine and agmatine were identified (Table S1) in rotifer eggs and were found in higher abundance in AM eggs in comparison with RE (*p* = 0.006; FC = 6.89). Agmatine serves as the precursor for putrescine, the latter of which is the diamine precursor of polyamine metabolism including spermidine and spermine. Higher dopamine concentrations in the hemolymph and central nervous system in the silkworm larvae and pupae, contribute to the induction of diapause by enhanced production or release of the diapause hormone^81^. Dopamine was only detected in AM eggs suggesting that either it functions only as neurotransmitter in rotifer embryos or that the induction potential of dormancy in rotifers is limited to AM embryo’s or adults.

In conclusion, the current metabolomics analyses provided a wealth of information on the abundance of a large number of features in rotifer embryos. Numerous features were assigned to putative functional pathways revealing differences between dormant embryos and non-dormant developing embryos in glycolysis, TCA cycle, amino acid biosynthesis, as well as purine and pyrimidine metabolic pathways. In fact, the results show on one hand a metabolically active egg (AM) and on the other hand an egg in full dormancy (RE) in which all biosynthetic pathways are turned off, including the production of ATP that is the canonical energy donor in a myriad of biosynthetic reactions. A large number of features with an association with these pathways were lower or were not detectable in RE when compared with AM. These suppositions are consistent and further support the conclusions reached from our previous proteome profiling studies^17^ and from numerous studies (mostly in insects but also in other organisms) on the “dormancy syndrome”^1,4,29,42,44,48,52,53^. In addition, the current study also highlights specific features in rotifers which possibly function in other aquatic dormant embryos. Surprisingly, most features were found at significantly higher levels in AM than in RE; these include carbohydrates with a function in resistance to desiccation, vitamins (except for riboflavin that was detected only in RE), fatty acids and lipid classes (except for phosphatidylglycerols, which were found in higher abundance in RE). The abundance of L-kynurenine, with an established function in blocking UV penetration and a possible function in hatching, was lower in RE. Our metabolomics results support the displayed phenotype and the common functional pathways that have been described in organisms exhibiting dormancy. As there is little similarity in the transcriptomic profiles among dormancies displayed by distinct species, the present study demonstrates large scale metabolomics as a promising research avenue for functionally comparing dormancy across species, including model and non-model organisms, since it is applicable without prior knowledge of the genomic sequence.

## Materials and Methods

### Rotifer cultures and collection of RE

#### Rotifer cultures

Rotifers (clone ATB4) were cultured at 24–26 °C in artificial sea water (Red Sea salts, Israel) at 40 ppt fed with concentrated algae (*Nannochloropsis sp*.) prepared from cultures at Maagan Fish Farms, Israel (samples used for ^1^H NMR and HPLC analyses, below) or from concentrated algae prepared by Galil Algae, Israel (samples used for LC-MS, MS/MS and GC-MS measurements, below). Rotifers were induced to produce RE by transferring them from high salinity (42.5 gm L^−1^; 40 ppt) to low salinity (10.75 gm L^−1^; 10 ppt) culture conditions and RE production commenced synchronously, 3–5 days after this transfer and RE were collected after 9–15 days of culture.

#### Collection of RE

Hydrated RE were collected from the bottom part of culture flasks, cleaned manually and transferred into Eppendorf vials, washed and frozen in liquid nitrogen and stored at −80 °C until analysis. Detailed description of the procedures for collection and counting of RE and AM is provided in Supplementary Information Text file 1. Lyophilized samples were shipped on dry ice to Leiden University, The Netherlands. Five samples from five cultures were collected with an estimated number of RE per sample ranging from 16,000 to 26,000. For LC-MS, MS/MS and GC-MS measurements (below), RE samples were frozen in liquid nitrogen and shipped to metaSysX, Germany on dry ice for metabolomics and lipidomics analyses. Three replicate samples from three different cultures, with an estimated number of 24,000 RE were used.

#### Collection of non-dormant AM

(a) For ^1^H NMR and HPLC analyses (below), amictic females were collected from 10 ppt sea water rotifer cultures that were maintained at similar conditions to RE producing cultures. AM were removed from females and collected into Eppendorf vials (detailed description of the procedure is provided in Supplementary Information Text File 1). The vials with the non-dormant eggs were frozen in liquid nitrogen, stored at −80 °C and lyophilized before shipping the samples on dry ice to Leiden University for metabolomics analysis. (b) For LC-MS, MS/MS and GC-MS measurements (below), rotifers were cultured for 2–3 days in seawater at 10 ppt. The collection and counting of AM eggs was performed as described in Supplementary Information Text File 1. The vials with nearly dry pellet were weighed and frozen in liquid nitrogen and shipped to metaSysX, Germany on dry ice. AM eggs (~24,000 per sample) from three different cultures were analyzed.

#### Samples of algae used as food in rotifer cultures

In order to distinguish between metabolites found in rotifers and those originating from algae, three replicate samples of concentrated algae (Galil Algae, Israel) were also collected, frozen in liquid nitrogen, stored at −80 °C and frozen samples were shipped on dry ice to metaSysX, Germany.

#### Determination of moisture content of RE and AM

Three samples of RE (weighing 0.1537, 0.1543 and 0.1354 g) were collected into 1.5 ml Eppendorf vials from three cultures (as described above) and similarly, three AM samples (weighing 0.2315, 0.2402 and 0.1534 g) were collected (as described above) from three cultures. Samples were weighed before and after incubation at 58 °C for 24, 48 and 96 hr. The dry weight did not change after 48 hr. The percent moisture was calculated according to: dry weight after 48 hr/wet weight × 100.

### Chemical analyses of samples

#### ^1^H NMR analyses

^1^H nuclear magnetic resonance (NMR) spectroscopy was used due to its wide range of metabolic coverage and its ease of quantification of the constituents. This was followed by high-performance liquid chromatography (HPLC) coupled to a diode array detector (DAD), for the targeted analysis, especially for biomarker pigments such as xanthophylls. Detailed description of the extraction methods for NMR analysis, analysis of ^1^H NMR results and HPLC sample preparation and analyses is provided in Supplementary Information Text File 2.

#### ^1^H NMR spectral data analyses

The NMR spectra were binned and subjected to multivariate analysis. Thereby, the shared and discriminant markers could be identified. For multivariate data analysis, ^1^H NMR spectra were scaled to total intensity and reduced to integrated regions of equal width (0.04 ppm) from 0.30–10.0 ppm and automatically binned by AMIX software (v.3.7, Biospin, Bruker). The regions of δ 4.70–5.00 and δ 3.28–3.34 were excluded from the analysis due to the residual signals of water and methanol, respectively. In order to cluster the samples, Principle Components Analysis (PCA) was performed with SIMCA-P software (version 12.0, Umetrics, Umeå, Sweden). Scaling was based on Pareto^82^.

#### LC-MS, MS/MS and GC-MS measurements as well as data processing, annotation and normalization

AM and RE were extracted according to a modified protocol previously described^83^. After extraction, the volume collected for further analysis was adjusted to the number of eggs (~24,000 eggs per sample). LC-MS, MS/MS and GC-MS measurements, data processing, annotation and normalization, are described in Supplementary Information Text File 2.

#### Statistical analysis of GC-MS

The total ion chromatograms showed large differences between the RE and AM samples. Two methods were used for analyses of data: (a) Median group-wise normalization where the median of log transformed intensities of each sample was first subtracted from the log transformed intensities of each feature and then the median of intensities of the whole group was added to the log transformed intensity of each feature (Table S1 and Fig. S3). This type of normalization does not remove the biological responses of the different group of samples but rather eliminates the systematic technical errors between the replicates within the same groups of samples caused, for example by different extraction efficiencies. The effect on the distribution of intensities is shown in Fig. S3. Also, this type of normalization will not influence the variation between the groups of samples and it will not change the biological differences especially between samples that are characterized by big differences in ion intensities. The normalized intensities were log_2_ transformed before performing student’s *t test* between the AM and RE samples. The *p*-values were adjusted with use of Benjamini-Hochberg (BH) correction procedure^84^. The fold-changes of all features were calculated by dividing mean of normalized intensity of AM samples by mean of normalized intensity of RE samples. Unless otherwise indicated, the Figures and Tables in the manuscript use the normalized dataset (Table S1), and (b) Data were not normalized (Table S2 and Fig. S6) and include all features that were identified. Zero intensities were replaced by missing values and thus neglected in the subsequent analyses. Non-zero intensities were Log2 transformed. A 1-Way ANOVA was carried out for each feature, for the experimental group effect (AM, RE, algae), including one contrast: AM vs RE. Log2 of the Fold Change of AM vs RE was compared to that of the normalized dataset (a) above). For the comparison of the Fold Change we only included features which had values in both datasets (Fig. S5). Features with FDR adjusted *p*-value < 0.05 for the contrast AM vs. RE (1,968 features) in the un-normalized data were submitted to hierarchical clustering in Partek Genomics Suite using Pearson’s dissimilarity and complete linkage (Fig. S6 and Table S3). Prior to clustering, intensities were standardized such that the mean and standard deviation per feature were 0 and 1, respectively.

## Supplementary information

List of Supplementary Files

Table S1

Table S2

Table S3

Table S4

## Data Availability

All data generated or analyzed during the current study are included in this published article and its Supplementary Information Files. Supplementary Information used and/or analyzed during the current study are available from the corresponding author on reasonable request.
