# Supplementary material for: Metabolomics reveals novel insight on dormancy of aquatic invertebrate encysted embryos
Source: Sci Rep. 2019 Jun 20;9:8878. doi: 10.1038/s41598-019-45061-x (PMC6586685; doi:10.1038/s41598-019-45061-x)

**Corresponding author: Esther Lubzens**

Title of manuscript: Metabolomics reveals novel insight on dormancy of aquatic invertebrate encysted embryos

**Authors**: Evelien Rozema^1*^, Sylwia Kierszniowska^2*^, Oshri Almog-Gabai^3^, Erica G. Wilson^1^, Young Hae Choi^4^, Robert Verpoorte^1^, Reini Hamo^3^, Vered Chalifa-Caspi^5^, Yehuda G. Assaraf^3,6^ & Esther Lubzens^3, &^

^1^Natural Products Laboratory, Institute of Biology, Leiden University, Leiden, The Netherlands. ^2^metaSysX GmbH, 14476 Potsdam-Golm, Germany. ^3^Faculty of Biology, Technion, Haifa, Israel. ^4^College of Pharmacy, Kyung Hee University, 02447, Seoul, Republic of Korea. ^5^National Institute for Biotechnology in the Negev, Ben-Gurion University of the Negev, Beer-Sheva, Israel. ^6^The Fred Wyszkowski Cancer Research Lab.

*Equal contribution

Correspondence and requests for materials should be addressed to E.L. (email: elubzens@technion.ac.il)

**List of Supplementary Files:**

**1. Text 1: Methods for collection and counting of resting eggs and amictic eggs**

**2. Text 2: Methods for NMR analysis and LC-MS, MS/MS and GC-MS measurements, data processing and data annotation**

**3. Figures S1- FigureS9; pdf format**

**4. Tables: Table S1- Table S4; XLs format**

**Supplementary Text File S1: Methods for collection and counting of resting eggs and amictic eggs**

*Collection of RE:* Hydrated RE were collected from the bottom part of culture flasks, cleaned manually using a compound microscope (Leica, Germany) and transferred into Eppendorf vials. The collected RE were centrifuged at room temperature [10 min at room temperature, 22-25⁰C; 2,300X g in a Spectrafuge 24D (Labnet)]. For ^1^H NMR and HPLC analyses the pellet containing the RE was briefly washed twice with double distilled water and a 10 µl aliquot was removed from each pellet to estimate the number of RE in each vial. Subsequently, the vials with RE were frozen in liquid nitrogen and stored at -80⁰C until analysis. Samples were lyophilized and shipped on dry ice to Leiden University, The Netherlands. This way, five samples from five cultures were collected with an estimated number of RE per sample ranging from 16,000 to 26,000. For LC-MS, MS/MS and GC-MS measurements, RE were suspended in 20 ml 10ppt seawater after the cleaning procedure and 3x100 μl samples were removed for counting RE in each sample. Afterwards, the content of the vial with RE was poured into a 35 mm glass petri-dish and RE were collected into a 1.5 ml vial. Vials with nearly dry pellet were weighed and frozen in liquid nitrogen. They were then shipped to metaSysX, Germany on dry ice for metabolomics and lipidomics analyses. Three replicate samples from three different cultures, with an estimated number of 24,000 RE were used.

*Collection of non-dormant AM:* a) For ^1^H NMR and HPLC analyses, amictic females (Clone ATB4) were removed from 10 ppt sea water rotifer cultures that were maintained at similar conditions to RE producing cultures, except that the density of rotifers was > 50-100 rotifers/ml, to reduce RE formation. The top layer of the culture was sieved through 200 μm mesh and then 60 μm, and re-suspended in 500 ml of 10 ppt seawater. This was repeated twice and the rotifers were suspended in a glass beaker in 60 ml seawater (10 ppt) and distributed to 50 ml Falcon vials (~15 ml in each vial). To remove the AM carried by females, the vials were agitated for 3 min at top speed on a vortex. The content of the vials was placed in a glass beaker and after 10-15 min, the top layer containing females was removed and the AM were collected from the bottom into a 35 mm glass plate. Subsequently, the AM were collected by using a compound microscope into sterile Eppendorf vials placed on ice. The collected eggs were centrifuged at 5,000 rpm for 3 min (Spectafuge 24D, Labnet International Inc.) and the pellet was washed briefly twice with sterile distilled water. The number of eggs was counted in 10 µl triplicate samples. The vials with the AM were frozen in liquid nitrogen, stored at -80°C and lyophilized before shipping the samples on dry ice to Leiden University for metabolomics analysis.

b) For LC-MS, MS/MS and GC-MS measurements, rotifers were cultured for 2-3 days in seawater at 10 ppt as described above, at a density ranging from 10-30 rotifers/ml. The short culture duration ensured that all females carried only AM. The collection of AM was performed as described above in (a). After the final cleaning step, AM were suspended in 20 ml of 10 ppt seawater in 50 ml Falcon vials and the number of RE was counted in 3x100 μl samples. The content of Falcon vial was poured into 35 mm glass plate and the AM were collected into Eppendorf vials placed on ice as described in (a) above. The AM were washed twice with sterile phosphate buffer solution (Sigma, pH=7.0) and centrifuged as described above. The vials with the nearly dry pellet were weighed and frozen in liquid nitrogen and shipped to metaSysX, Germany on dry ice. RE from three different cultures were analyzed.

**Supplementary File Text 2: Methods for NMR analysis and LC-MS, MS/MS and GC-MS measurements, data processing and data annotation**

*Extraction for NMR analysis:* A few milligrams of lyophilized rotifer egg material were weighed depending on the yield into 2 mL microtube and extracted at 1 mg/100 μL NMR solvent. The NMR solvent consisted of a mixture (1:1, v/v) of methanol-d_4_ (99.8% % atom D from Cambridge Isotope Laboratories, Tewksbury, MA, USA), and phosphate (KH_2_PO_4_) buffer at pH 6.0 in deuterium oxide (> 99.9% from CortecNet, Voisins-Le-Bretonneux, France) containing 0.01% 3-(trimethylsilyl)propionic-2,2,3,3-d_4_ acid sodium salt (TSP), (98 % atom D from Sigma-Aldrich) (2010 (TSP, w/w) as an internal standard for calibration of chemical shift. Samples were ultrasonicated for 5 min (Branson Ultrasonics, USA; level used was 40 kHz) followed by centrifugation at 13,000 rpm for 10 min. The supernatant was collected and transferred to 3 mm NMR tubes for ^1^H NMR analysis.

*Analysis of ^1^H NMR results:*  ^1^H NMR measurements were carried out as previously described^1^. ^1^H NMR spectra were recorded with a Bruker AV 600 spectrometer (Bruker, Karlsruhe, Germany). For each sample, 64 scans were recorded. FIDs were Fourier transformed with LB = 0.3 Hz. Manual phase adjustment and baseline correction were applied as well as calibration with TSP as standard to 0 ppm using Topspin software (Bruker, Karlsruhe, Germany). For identification of the signals, the in-house reference database and literature references were used.

*HPLC sample preparation and analyses:* A few milligrams of lyophilized rotifer egg sample were weighed depending on the yield into 2 mL microtube and 1 mg sample was extracted with 100 μL acetone: double distilled water (v/v 9:1) and ultrasonicated for 5 min and subsequently the sample was filtered through a 0.2 micron RC membrane filter (Minisart RC4, Sartorius, Germany). The filtered extract was transferred to a brown HPLC vial and HPLC analysis was performed using an Agilent Technologies 1200 Series high performance liquid chromatography system (Agilent, Folsom, CA, USA), equipped with a Diode Array Detector G13150 and autosampler. A Luna C18 column (5 μm particle size, 250 × 4.6 mm i.d.) (Phenomenex, Torrance, CA, USA) was equipped with a guard column and column temperature was set at 25°C. Ten μL of sample was injected for analysis. A gradient was applied with acetonitrile: water (9:1, v/v) as solvent A and 0.5 M of ammonium acetate in water as solvent B according to the following scheme with a flow rate of 0.9 mL/min: t=2.0 min A=100%; 2.4 min A=90%, B=10%; t=10.0 min A=65%, B=35%; t=17.0 min, A=31%, B=69%; t=18.0 min A=100%, B=0% and 2 minutes hold. Quantitative measurements were performed at a wavelength of 440 nm.

LC-MS, MS/MS and GC-MS measurements, data processing, data annotation and data normalization: AM and RE were extracted according to a modified protocol previously described^2^. After extraction, the volume collected for further analysis was adjusted to the number of eggs (~24,000 eggs per sample). LC-MS, MS/MS and GC-MS measurements, data processing, annotation and normalization, are described below.

*LC-MS Measurements (Hydrophilic and Lipophilic analytes):* The samples were measured with the use of a Reversed Phase Ultra Performance Liquid Chromatography (RP-UPLC, Waters ACQUITY) coupled to a mass spectrometer which consists of an electrospray ionization source (ESI) and an Orbitrap-type mass analyzer (Thermo-Fisher, Bremen, Germany). Chromatographic separation was performed on a Reversed Phase Bridge Ethyl Hybrid (BEH) C_8_ and a Reversed Phase High Strength Silica (HSS) C_18_ column (Waters) for lipophilic and hydrophilic compounds, respectively. The mobile phase composition used for separation was previously described^2^. The mass spectra were acquired in survey full scan MS in positive and negative mode (Mass Range [100-1500]) for polar and lipophilic analytes. Additionally, the lipophilic compounds were measured in dd-MS/MS (data dependent tandem acquisition mode) collision energy 40 eV) in positive and negative mode in mass range (100-1500).

*LC-MS Data Processing (Hydrophilic Analytes):* The data analysis was performed with the software REFINER MS® 10.0 (GeneData, <http://www.genedata.com>). Alignment and filtration of the LC-MS data were completed using in-house R-based software. After extraction of the peak list from the chromatograms, the data were processed, aligned and filtered. At this stage an average RT and an average *m*/*z* values were given to the features. Thereafter, isotopic peaks, in-source fragments and lower intensity adducts were removed from the data. The annotation of the content of the sample was accomplished by matching the extracted data from the chromatograms with our library (metaSysX GmbH, Potsdam, Germany) of reference compounds. For quantification of metabolites, the most intense adduct was used.

*GC-MS Measurements:* Sample derivatization and analysis were carried out as described previously^3^. The GC-MS analysis was conducted using an Agilent Technologies GC coupled to a *Leco Pegasus HT* mass spectrometer, which consists of an EI ionization source and a time-of-flight (TOF) mass analyzer. Sample volumes of 1 μl were injected into the GC column: 30 meters DB35; Starting temp: 85°C for 2 min; Gradient: 15°C per min up to 360°C.

*Data analyses, GC-MS Data Processing and Annotation*: NetCDF files from the Leco Pegasus software were imported to R program. The Bioconductor package TargetSearch^3^ was used to transform retention time to retention index (RI), to align the chromatograms, to extract the peaks, and to annotate them by comparing the spectra and the RI to the Fiehn Library and to a user created library. Annotation of peaks was manually confirmed in Leco Pegasus. Analytes with similar RI and identical mass were relatively quantified. Metabolites with a RT and a mass spectrum that did not result in a match in the databases were labeled as unknown metabolites.

**Cited References**

1. Kim, H. K., Choi, Y. H.& Verpoorte, R. NMR-based metabolomic analysis of plants. *Nat. Protoc.* **5**, 536–549 (2010).2. Giavalisco, P., Köhl, K, Hummel, J., Seiwert, B. & Willmitzer, L. 13C isotope-labeled metabolomes allowing for improved compound annotation and relative quantification in liquid chromatography-mass spectrometry-based metabolomic research. *Anal. Chem.* **81**, 6546-6551 (2009).

3. Lisec, J., Schauer, N., Kopka, J., Willmitzer, L & Fernie, A. A. Gas chromatography massspectrometry-based metabolite profiling in plants. *Nat. Protocol*. **1**, 387-396 (2006).

4. Cuadros-Inostroza, A. *et al.* TargetSearch - a Bioconductor package for the efficient pre- processing of GC-MS metabolite profiling data. *BMC Bioinformatics* **10**, 428 (2009).

**List of Supplementary Files Figures:**

**Figure S1:** The outline of this study.

**Figure S2:** Representative metabolic profiles of AM and RE. **(1)** 600 MHz ^1^H nuclear magnetic resonance (NMR) spectra of the crude extracts of RE (**a**) and AM (**b**), respectively. In general, the levels of metabolites are higher in AM when compared to RE. The expansions of the spectra (**a** and **b**) are shown in **c, d, e, f** and **g** in each range**.** The expansions in the range of δ 0.8–δ 3.3 for RE and AM are shown in **c** and **d**, respectively. The expansion in the range of δ 3.3–δ 5.3 for RE and AM are shown in **e** and **f**, respectively. The expansion in the range of δ 5.7–δ 8.7 for RE is shown in **g**. TSP is the internal standard. 1. valine, 2. ethanol, 3. CH_2_ signals of lipids, 4. lactate, 5. alanine, 6. acetate, 7. 1,3-diaminopropane, 8. homoserine, 9. proline, 10. acetone,11. glutamic acid, 12. trimethylamine, 13. betaine, 14. trehalose, 15. glycine, 16. creatine-like compound, 17. residual water, 18. AMP, 19. (phenyl)alanine, 20. histamine, 21. xanthine, 22. Oxipurinol.. **(2)** HPLC chromatograms with detection at 440 nm of AM (**h**) and RE (**i**). In the AM, two major xanthophylls were detected with retention times 10.5 min and 11.1 minutes in low quantities that were not further identified.

**Figure S3:** A box-plot of the intensity distribution (Log2) in all samples before (a) and after (b) normalization.

**Figure S4:** Distribution of un-normalized intensities of all features (a) and annotated features (b) of Table S2.

**Figure S5:** Comparison between the fold-change values of AM versus RE in the un-normalized dataset (x-axis) and the normalized dataset (y-axis) of all features.

**Figure S6**: Hierarchical clustering showing the relative abundance of features in algae, AM and RE. Only features with FDR adjusted p-value < 0.05 in the comparison AM vs. RE (using un-normalized data) are shown. Missing values are shown as white lines. For details see Table S3.

**Figure S7:** A partial KEGG map^1^ (<https://www.kegg.jp/kegg-bin/show_pathway?map00380>) for tryptophan metabolism highlighting the proteins that are associated with L-kynurenine metabolism in rotifer eggs. A rectangle with a yellow background indicates a protein that was detected only in AM. A rectangular or circle with a red background indicates a protein with a lower abundance in RE. A rectangular or a circle with a blue background indicates a protein with a higher abundance in RE. Protein abundance is taken from [17].

**Figure S8**: A KEGG map^1^ (<https://www.kegg.jp/kegg-bin/show_pathway?map00230>) for Purine metabolism showing the abundance of RE and AM proteins and relative intensities for features. A rectangle or a circle with a yellow background indicates that the protein or a feature, respectively, was detected only in AM. A rectangle or circle with a red background indicates that the abundance of a protein or the relative intensity of a feature, respectively, was lower in RE. A rectangle or a circle with a blue background indicates that the abundance of a protein or the relative intensity of a feature, respectively, was higher in RE. A rectangle with a gray background indicates the detection of a protein, but a similar abundance or relative intensities in AM and RE. A circle with a black background indicates that the feature was detected with similar abundance in AM and RE. Protein abundance is taken from a previous paper [17].

**Figure S9**: A KEGG map^1^ (<https://www.kegg.jp/kegg-bin/show_pathway?map00240>)

for Pyrimidine metabolism showing the abundance of RE and AM proteins and relative intensities for features.. A rectangle or a circle with a yellow background indicates that the protein or a feature, respectively, was detected only in AM. A rectangle or circle with a red background indicates that the abundance of a protein or the relative intensity of a feature, respectively, was lower in RE. A rectangle or a circle with a blue background indicates that the abundance of a protein or the relative intensity of a feature, respectively, was higher in RE. A rectangle with a grey background indicates the detection of a protein, but a similar abundance or relative intensities in AM and RE. A circle with a black background indicates that the feature was detected with similar abundance in AM and RE. Protein abundance is taken from a previous paper [17].

**Supplementary Files Tables:**

**Table S1:** A list of the identified (all) and annotated features by LC-MS, MS/MS and GC-MS measurements in algae, RE and AM. The normalized intensities were log_2_ transformed before performing student’s *t test* between the AM and RE samples. The *p*-values were adjusted with use of Benjamini-Hochberg (BH) correction procedure. The fold-changes of all features were calculated by dividing mean of normalized intensity of AM samples by mean of normalized intensity of RE samples.

Columns: mz_diff_ppm provides information about the difference (expressed in ppm) between the measured *m*/*z* and the *m/z* measured for each reference compound analyzed and incorporated into the in-house database. Each column contains relative intensities of metabolite (feature) measured in the sample and logarithmically transformed intensities in corresponding columns with “log2” in the name.

**Table S2:** Un-normalized intensities for all features and comparison of AM vs. RE in a 1-Way ANOVA model for the experimental group effect (AM, RE, algae). Features with FDR adjusted p-value < 0.05 in the contrast AM vs. RE were considered “differentially abundant”. Sheet 1 – all features. Sheet2 – Differentially abundant features.

**Table S3:** Results of hierarchical clustering of differentially abundant features from Supplementary Table S2 (un-normalized data), along with their raw intensities and 1-Way ANOVA results.

**Table S4**: Lists of features that were identified only in RE or only in AM. The list is based on data shown in Table S1.

**Cited reference:**

1 Kanehisa, M.,Sato, Y., Furumichi, M., Morishima, K. & Tanabe, M. New approach for understanding genome variations in KEGG, Nuc. Acids Res. **47**, D590–D595 (2019), [doi.org/10.1093/nar/gky962](https://doi.org/10.1093/nar/gky962).

Fig S1


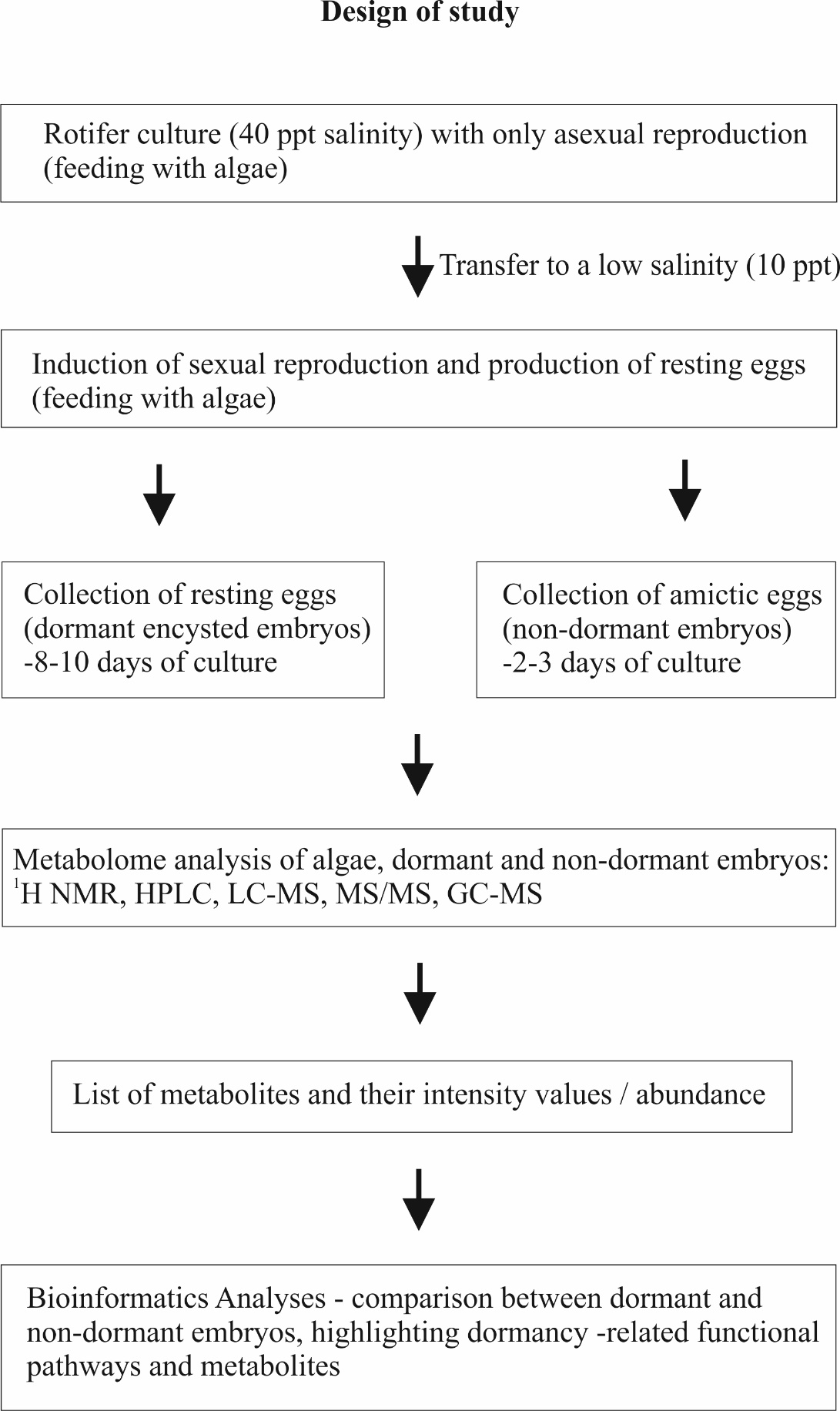


Fig S2


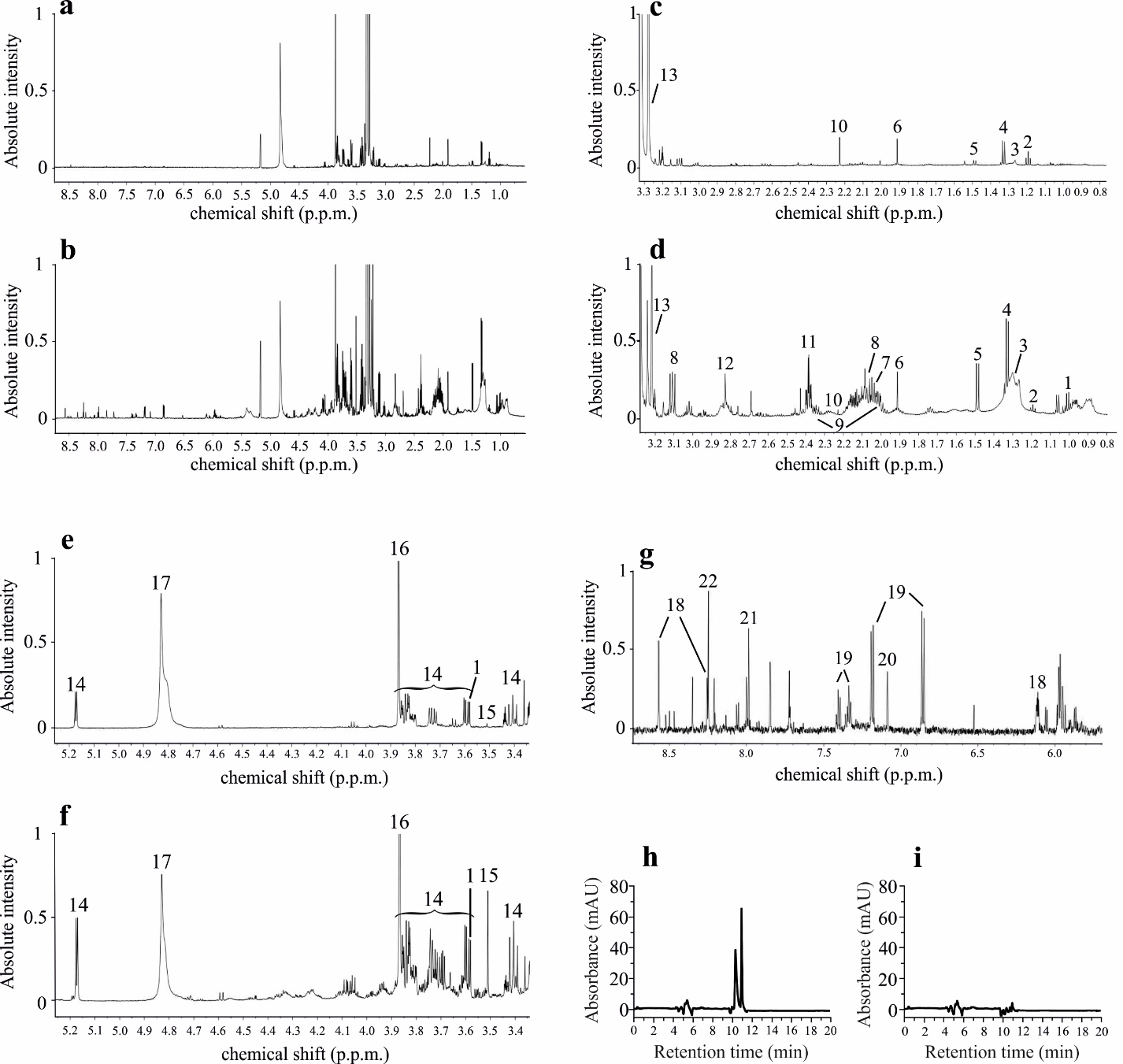


Fig S3


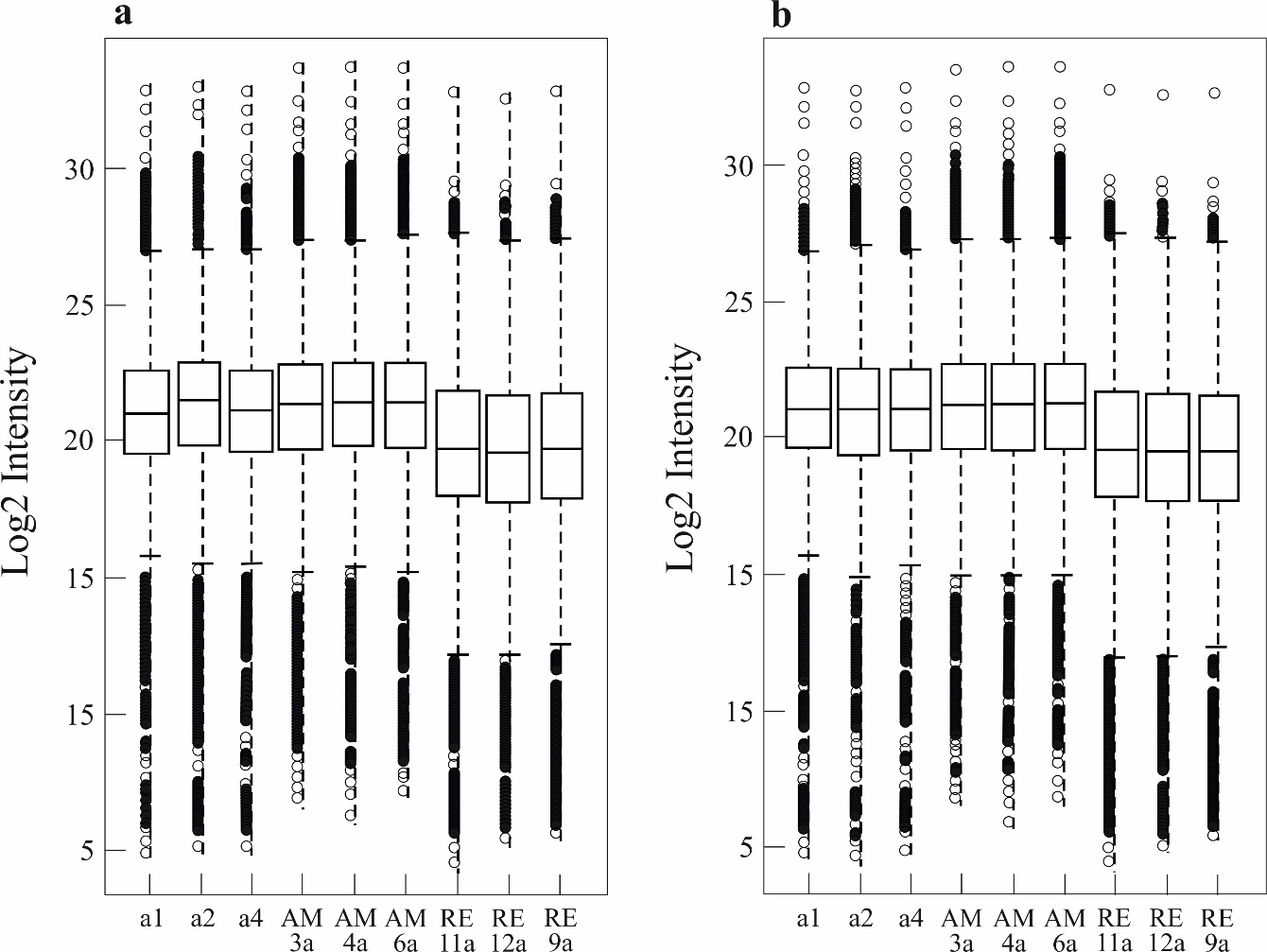


Fig S4


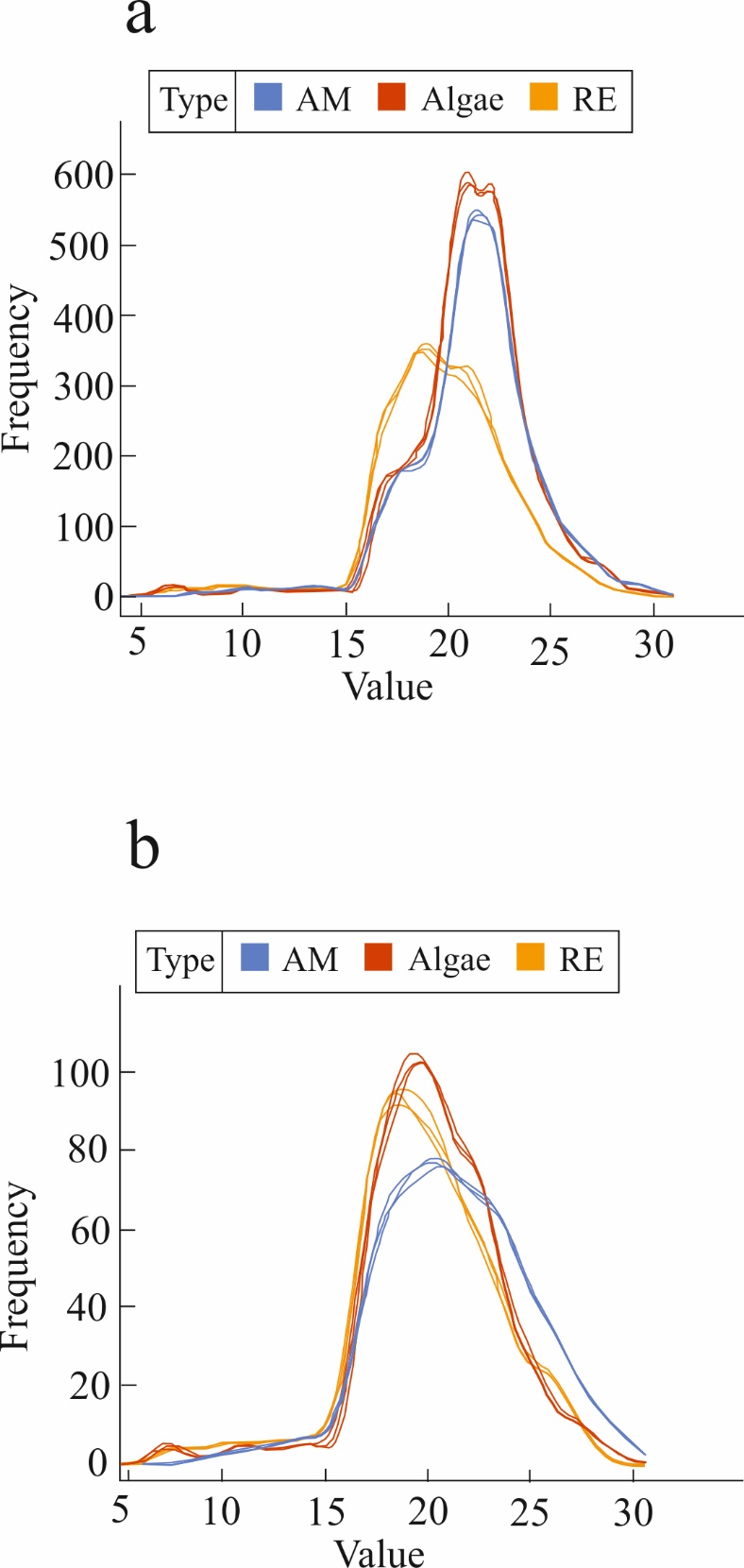


Fig S5


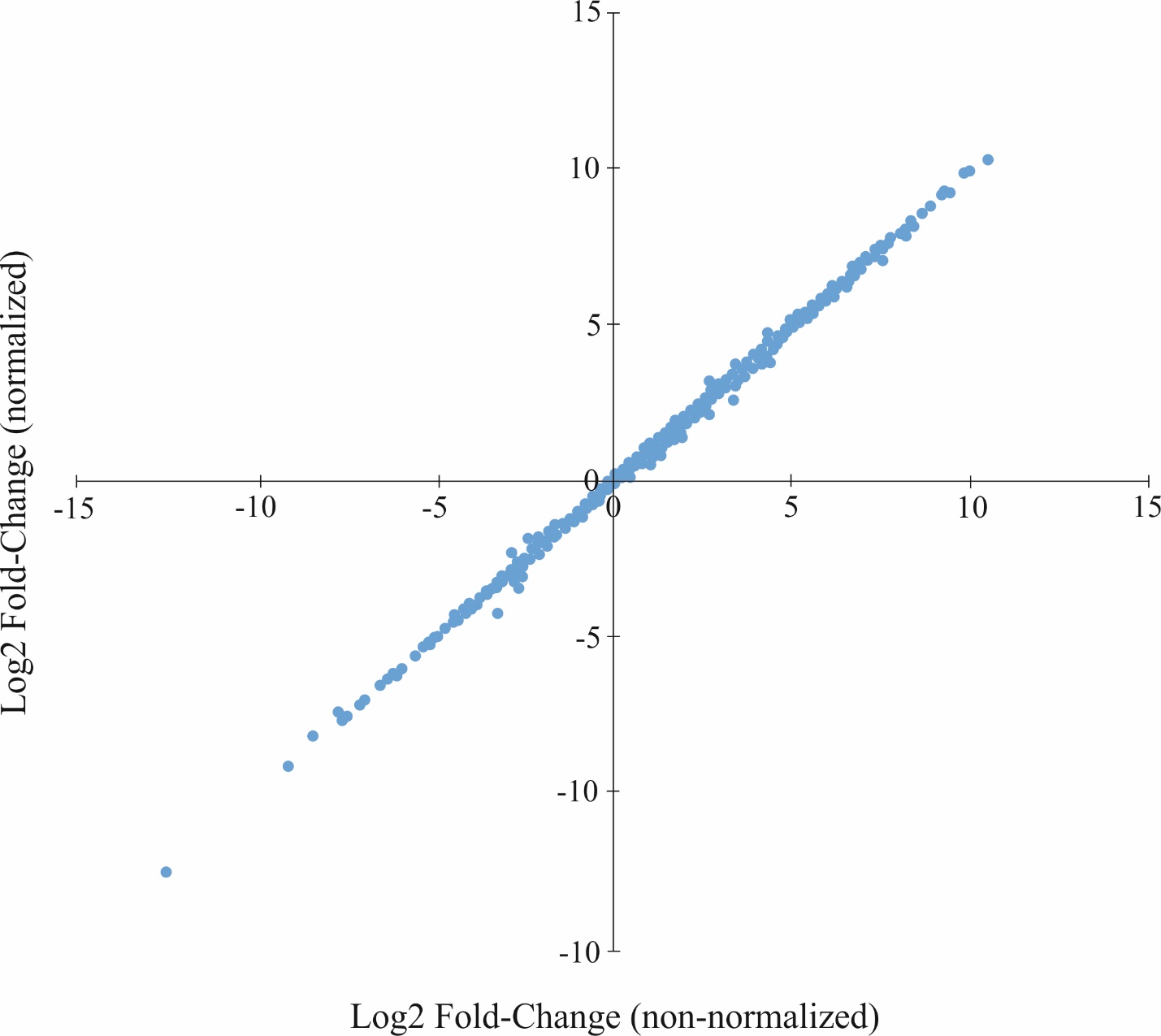


Fig S6


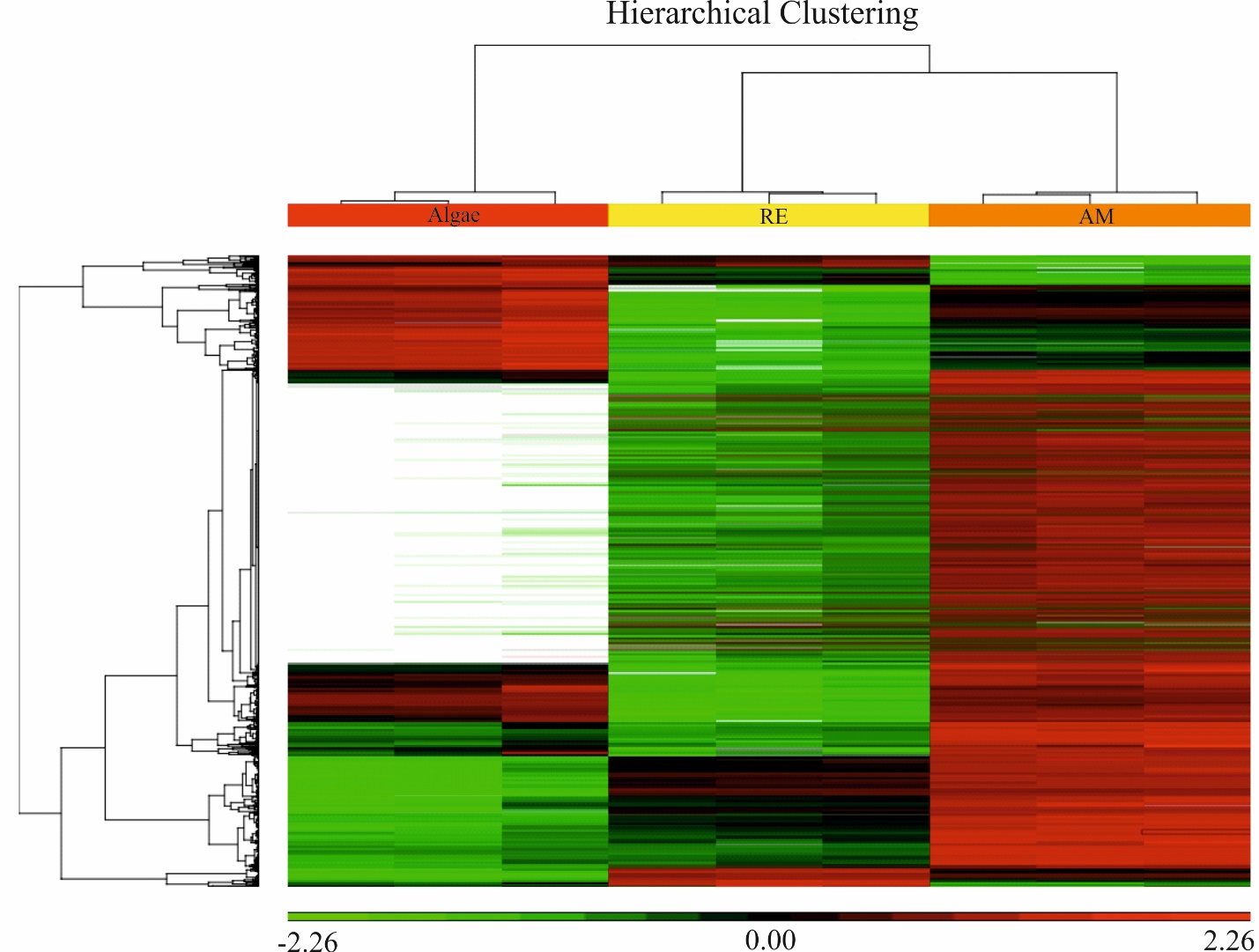


Fig S7


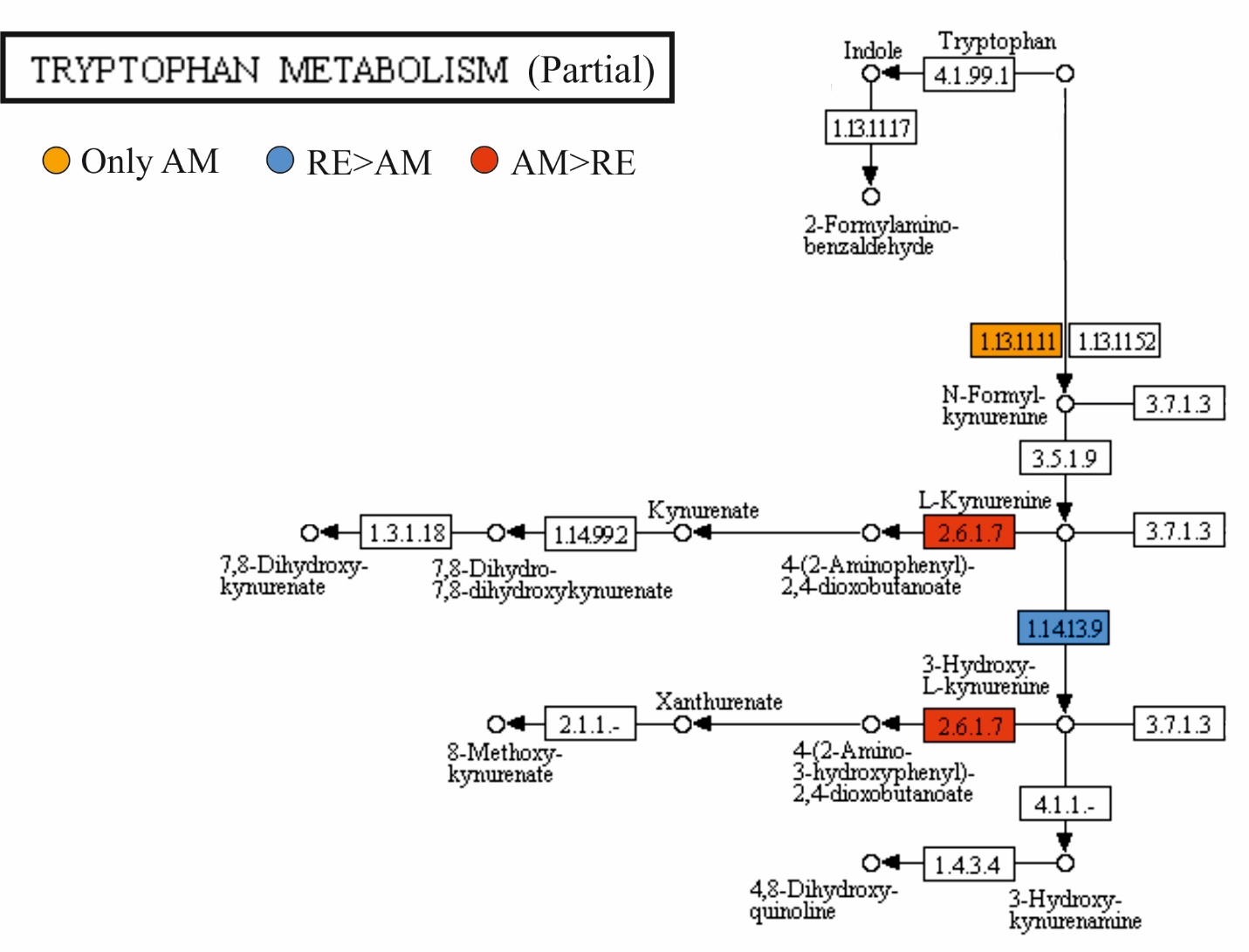


Fig S8


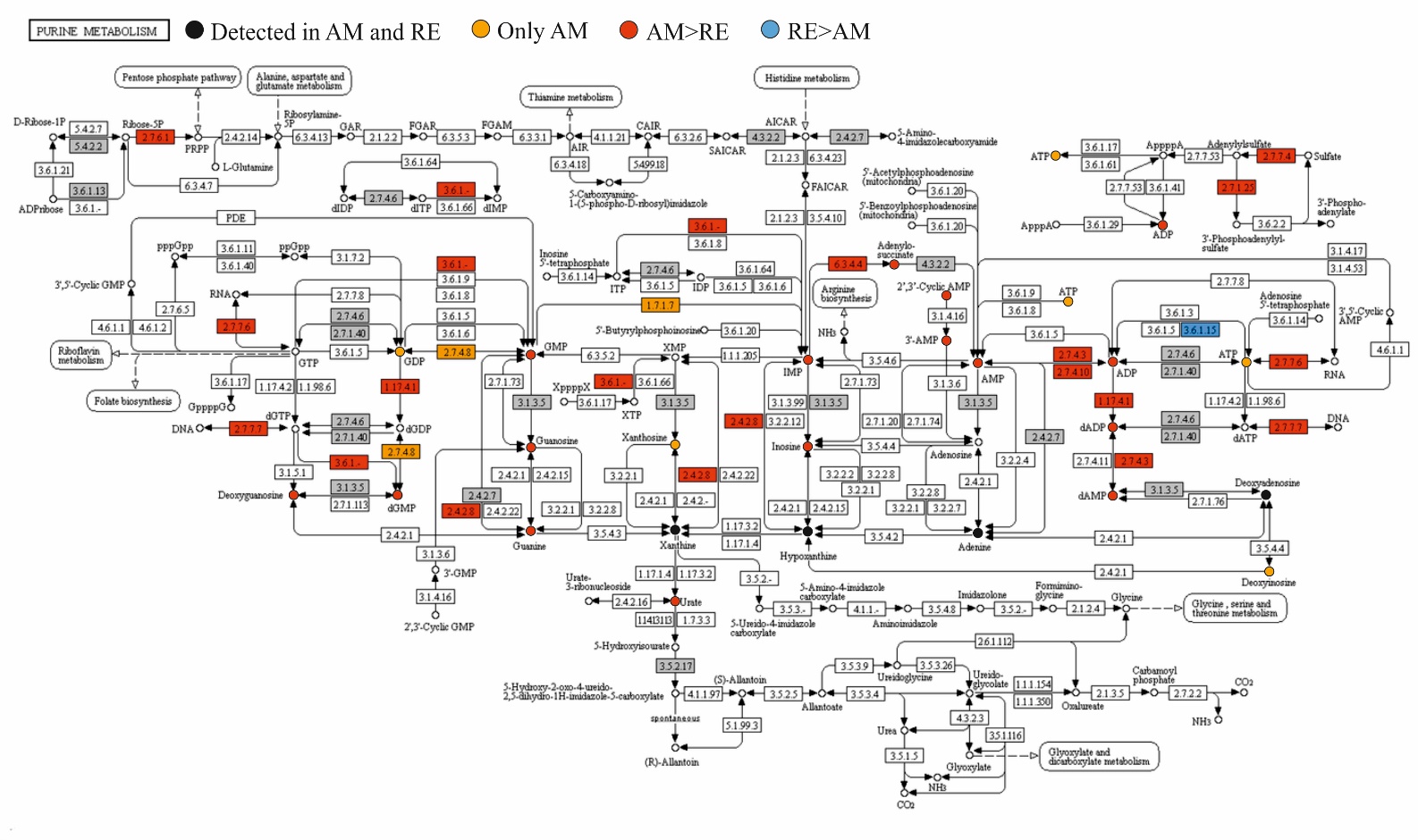


Fig S9


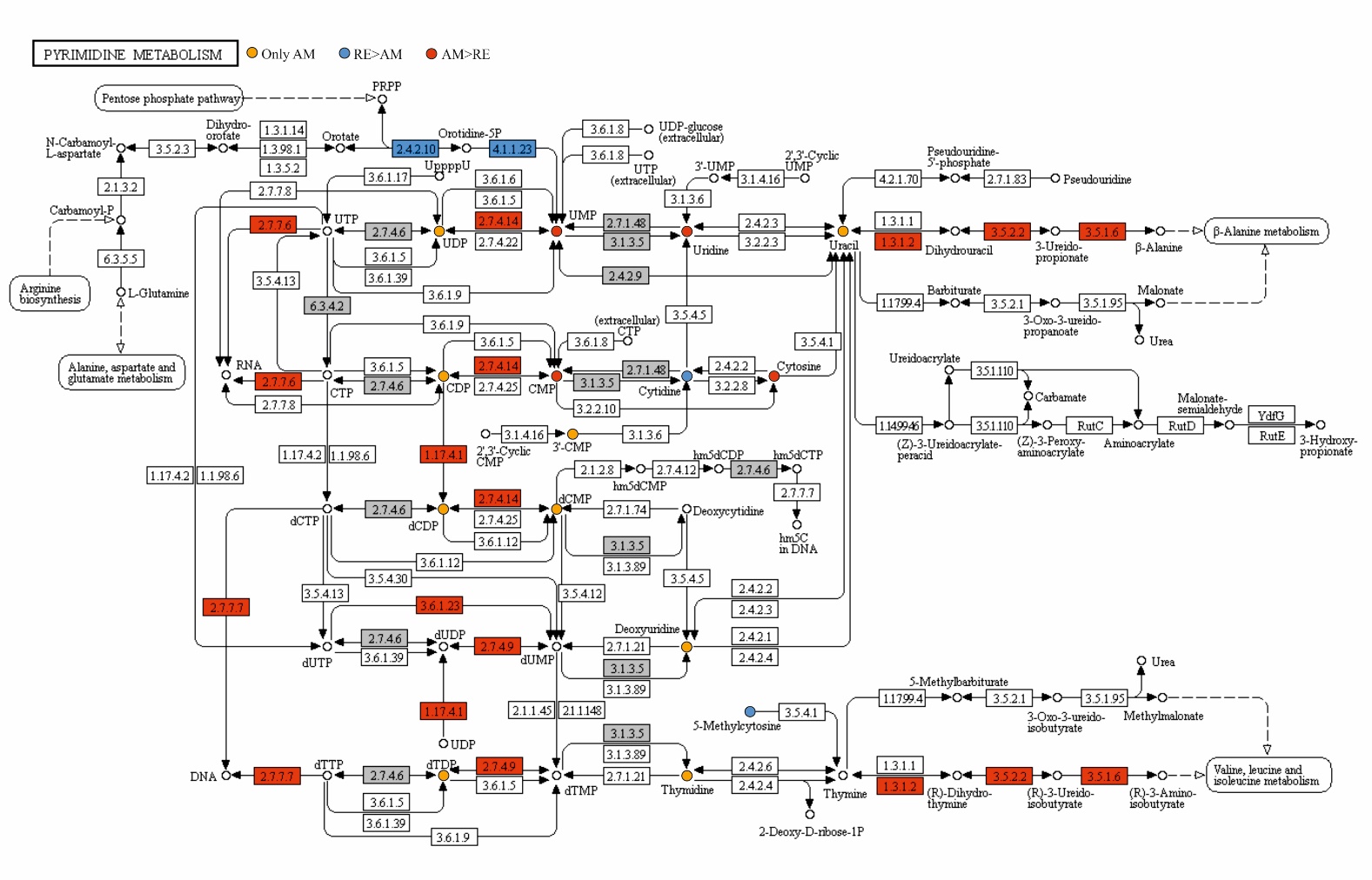

Supplement: Supplementary file 1 — List of Supplementary Files [file 41598_2019_45061_MOESM1_ESM.docx]
